# Signatures of increasing environmental stress in bumblebee wings over the past century: Insights from museum specimens

**DOI:** 10.1111/1365-2656.13788

**Published:** 2022-08-17

**Authors:** Andres N. Arce, Aoife Cantwell‐Jones, Michael Tansley, Ian Barnes, Selina Brace, Victoria E. Mullin, David Notton, Jeff Ollerton, Emma Eatough, Marcus W. Rhodes, Xueni Bian, James Hogan, Tony Hunter, Simon Jackson, Ashleigh Whiffin, Vladimir Blagoderov, Gavin Broad, Steve Judd, Phaedra Kokkini, Laurence Livermore, Mahika K. Dixit, William D. Pearse, Richard J. Gill

**Affiliations:** ^1^ Georgina Mace Centre for The Living Planet, Department of Life Sciences Silwood Park, Imperial College London Ascot UK; ^2^ School of Engineering, Arts, Science & Technology University of Suffolk Ipswich UK; ^3^ Department of Zoology University of Oxford Oxford UK; ^4^ Department of Earth Sciences Natural History Museum London UK; ^5^ Smurfit Institute of Genetics Trinity College Dublin Dublin Ireland; ^6^ National Museum Scotland Edinburgh UK; ^7^ Faculty of Arts, Science and Technology University of Northampton Northampton UK; ^8^ Oxford University Museum of Natural History Oxford UK; ^9^ World Museum Liverpool UK; ^10^ Tullie House Museum and Art Gallery Trust Cumbria UK; ^11^ Ipswich Museum (Colchester and Ipswich Museums) Ipswich UK

**Keywords:** *Bombus*, climate change, digitisation, entomological collections, fluctuating asymmetry, landmarking, pollinators, Procrustes

## Abstract

1. Determining when animal populations have experienced stress in the past is fundamental to understanding how risk factors drive contemporary and future species' responses to environmental change. For insects, quantifying stress and associating it with environmental factors has been challenging due to a paucity of time‐series data and because detectable population‐level responses can show varying lag effects. One solution is to leverage historic entomological specimens to detect morphological proxies of stress experienced at the time stressors emerged, allowing us to more accurately determine population responses.
2. Here we studied specimens of four bumblebee species, an invaluable group of insect pollinators, from five museums collected across Britain over the 20th century. We calculated the degree of fluctuating asymmetry (*FA*; random deviations from bilateral symmetry) between the right and left forewings as a potential proxy of developmental stress.
3. We: (a) investigated whether baseline *FA* levels vary between species, and how this compares between the first and second half of the century; (b) determined the extent of *FA* change over the century in the four bumblebee species, and whether this followed a linear or nonlinear trend; (c) tested which annual climatic conditions correlated with increased *FA* in bumblebees.
4. Species differed in their baseline *FA*, with *FA* being higher in the two species that have recently expanded their ranges in Britain. Overall, *FA* significantly increased over the century but followed a nonlinear trend, with the increase starting *c*. 1925. We found relatively warm and wet years were associated with higher *FA*.
5. Collectively our findings show that *FA* in bumblebees increased over the 20th century and under weather conditions that will likely increase in frequency with climate change. By plotting *FA* trends and quantifying the contribution of annual climate conditions on past populations, we provide an important step towards improving our understanding of how environmental factors could impact future populations of wild beneficial insects.

## Graphical abstract

Determining stress events improves our understanding of the drivers of population declines, but a lack of baseline data makes this challenging. Leveraging museum specimens, the authors measured the change in bumblebee wing‐shape fluctuating asymmetry (*FA*; proxy of stress) between 1900 and 2000. *FA* increased over the century, with warmer, wetter conditions predicting higher *FA*.

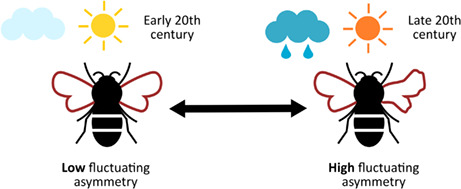

## 1 INTRODUCTION

Environmental change over the past century has placed multiple pressures on biodiversity, threatening important functional groups of organisms (Carmona et al., 2021; De Palma et al., 2016; Grab et al., 2019; Lenzen et al., 2012; Tilman et al., 2017; Trisos et al., 2020). Especially worrying have been the reports of declining insect pollinators (Forister et al., 2021; Nieto et al., 2014; Powney et al., 2019; Zattara & Aizen, 2021), as the vast majority of angiosperms (Ollerton et al., 2011) including >75% of the world's leading food crops (Klein et al., 2007) are to some degree dependent on their pollination service. Ultimately, if pollinators disappeared, half of the *c*. 350,000 species of flowering plants would lose an estimated ≥80% of their seed production, with a third of flowering plants estimated to fail to produce any (Rodger et al., 2021). Thus, distinguishing which factors are contributing to insect‐pollinator losses remains a research priority, and will help inform safeguarding strategies and predict future pollination services. Several global factors are associated with insect‐pollinator losses, including climate change, agricultural intensification and spread of invasive species (Ollerton, 2021; Potts et al., 2016). However, we are still limited in our ability to quantify the degree to which such factors have contributed to stress being placed on wild populations (Gill et al., 2016; Ollerton et al., 2014); defined here as a reduction in an individual's energy allocation towards development and reproduction (Beasley et al., 2013). We have typically been constrained to making inferences about past responses of wild populations based on occupancy modelling (Casey et al., 2015; Schleuning et al., 2016), laboratory studies (e.g. Kenna et al., 2021; Martinet et al., 2021; Smith et al., 2020) or snapshot comparisons of populations between past and contemporary landscapes (Carvell et al., 2017; De Palma et al., 2016). But this often does not account for the fact that the magnitude of these environmental stressors has varied historically in space and time. Part of the challenge of quantifying past stress is that time‐series data spanning the past century and beyond are scarce, stifling progress in understanding when historic insect‐pollinator populations actually experienced stress. Moreover, without comprehensive temporal data and a baseline understanding of stress experienced by insect‐pollinator populations, it is difficult to place any change into an appropriate historical context. A solution to help fill this data gap is to leverage museum specimens, which represent an underutilised biological data repository, to investigate how historic populations have responded to stressors (Freedman et al., 2020; Heberling, 2020; Oliveira et al., 2016; Owens et al., 2020; Scheper et al., 2014). By combining collection data across multiple museums, we can also overcome spatiotemporal biases in sampling (Wildman et al., 2022).

Mapping when and where stress has occurred is complicated, given that different stressors can impose varying lag effects on population sizes and geographic distributions (Wearn et al., 2012). This makes associating population declines or range contractions with historic environmental change challenging. An alternative approach is to use a biomarker or morphological signature as a proxy for levels of stress at the time environmental pressures were experienced, thereby enabling a more accurate assessment of the effects of such stressors. One such biomarker and potential proxy of stress is fluctuating asymmetry (*FA*), which are deviations from the bilateral symmetry of body plans (Palmer, 1994), reflecting developmental stability. Developmental stability refers to the ability of an organism to produce a consistent phenotype under different conditions (Palmer, 1994). As the left and right sides of bilaterally symmetrical traits are determined by the same genes, deviations from perfect symmetry are thought to be due to exogenous factors affecting the developmental process, for example the production of stress‐induced hormones (Benderlioglu, 2010). Although random deviations from asymmetry are expected in traits under normal conditions, high *FA* or increasing levels of *FA* are thought to reflect an inability to buffer development against suboptimal conditions, such as those induced by environmental change (Freeman et al., 1993; Palmer, 1994; Palmer & Strobeck, 1986). Directional changes in the levels of *FA* over time could therefore indicate that the underlying drivers of *FA* (that we interpret here to be ‘stressors’) are also changing.

Overall, *FA* has been suggested to increase in a diverse range of taxa (from butterflies to lizards to rodents; Table S1) in response to temperature (Gerard et al., 2018; Hosken et al., 2000; Imasheva et al., 1997; Nishizaki et al., 2015; Trotta et al., 2005), nutritional stress (Imasheva et al., 1997; Talloen et al., 2004), pesticide exposure (Abaga et al., 2011; Costa & Nomura, 2016; Friedli et al., 2020; Gerard et al., 2018; Simbula et al., 2021), infections and parasite load (Arundell et al., 2019; Bonn et al., 1996), urbanisation (Lazić et al., 2013; Leonard et al., 2018) and heavy metal pollution (Al‐Shami et al., 2011; Graham et al., 1993; Groenendijk et al., 1998), among other stressors (although see Bjorksten et al., 2001; Servia et al., 2004; Ward et al., 1998, who did not find this). For example, bumblebee *Bombus terrestris* wings have been shown to become less symmetrical under temperature (33°C) or parasitic stress (Gerard et al., 2018), as do honeybee *Apis mellifera* wings when individuals are exposed to pesticides (Friedli et al., 2020). Although some studies have not detected a link between *FA* and stress (e.g. Bjorksten et al., 2001; Floate & Fox, 2000), a meta‐analysis found stressors explained 36% of variation in *FA* across insect studies and concluded *FA* is a sensitive biomarker (Beasley et al., 2013). Indeed, *FA* has been observed to increase under even ‘natural’ environmental gradients, such as elevation (with butterflies: Henriques & Cornelissen, 2019), length of snow cover (Alpine chamois: Chirichella et al., 2020) and increasing summer temperatures (birds: Møller et al., 2018). Determining how environmental change could affect developmental stability is needed, because individuals with reduced developmental stability could have lower survival probability (e.g. Stringwell et al., 2014; Tocts et al., 2016; but see Clarke, 1995); yet, to our knowledge, long‐term studies investigating changes in *FA* are lacking.

Here we present a study using historical collections from a network of British museums to investigate changing *FA* as a signature of stress experienced by four bumblebee species (*Bombus hortorum*, *B. lapidarius*, *B. muscorum* and *B. pascuorum*) within Britain throughout the 20th century. Bumblebees are globally important pollinators (Goulson, 2010), and considered sensitive to environmental change (e.g. Settele et al., 2016), making them an important functional group to study. These four species were chosen as their distributions across Britain have changed differently over the past century: *B. hortorum* and *B. lapidarius* have maintained a relatively stable geographic distribution since the 1960s; *B. muscorum* has declined; and *B. pascuorum* expanded its distribution (Casey et al., 2015). To quantify levels of *FA* in these historic bumblebee populations, we landmarked photographs of forewings and collected label metadata for >3,300 specimens from five museum collections. After conducting a set of rigorous filtering steps (see Section 2) to consider only high‐quality specimens, we investigated: (a) if the amount of *FA* varies between species, and whether higher or lower *FA* is associated with distribution change over the century (described above); (b) if *FA* is higher in the second half of the 20th century given anthropogenic impacts on the environment have increased over the century, and whether change has shown a steady and consistent trend (linear relationship) or a more dynamic trend (nonlinear); and (c) if there is a correlation between *FA* and the climatic variables, mean annual temperature and precipitation, given both have been implicated in impacting bumblebee biology.

## 2 MATERIALS AND METHODS

We first photographed the museum specimens of the four bumblebee species and extracted corresponding metadata (such as collection location) from each label. We summarised wing shape by manually landmarking the left and right forewings, and used the difference in the left and right wing shapes to estimate *FA*. Next, we took a conservative approach to specimen inclusion by applying several data‐filtering steps (detailed below), to exclude specimens that might have artificially high *FA* (e.g. through having tilted wings as a consequence of the curatorial process). Additionally, to ensure an even distribution of data across the 20th century, we rarified our data (also detailed below). We did not require ethical approval for this study.

### 2.1 Initial dataset

Bumblebee collections were from Natural History Museum (London), National Museums Scotland (Edinburgh), Oxford University Museum of Natural History, Tullie House Museum and Art Gallery Trust (Carlisle) and World Museum (Liverpool), and included the species *Bombus hortorum*, *B. lapidarius*, *B. pascuorum* and *B. muscorum*. We photographed 6,311 specimens using either a Canon EOS 750D camera with a Canon Ultrasonic 100 mm macro lens and a Canon Macro Ring Lite MR‐14EX II flash or following Blagoderov et al. (2017), viz. using Canon EOS 550D and 700D cameras with custom‐built light boxes with 32W Circline VLR Full Spectrum Vita‐Lite 5500K fluorescent ring bulbs. From these, we extracted sample metadata from the label images, by transcribing collector name, collection date, location and caste, where present. All specimens had been collected throughout Britain and geolocating of specimens was carried out using Google Maps' Geocoding application programming interface, which returned a formatted address, country of origin and latitude and longitude coordinates (of the location's centroid using Mercator projection) for each specimen, using R code adapted from Lynn (2014). Location precision ranged from country to post code (Figure S1), with 66.6% of specimens located to city level (‘locality’) or higher resolution.

To associate each specimen with climatic data, we grouped locations into UK Met Office (2020) ‘climate regions’ (East Anglia; east and northeast England; northwest England and north Wales; southeast and central England; Midlands; south Wales and southwest England; east Scotland; north Scotland; west Scotland). Climatic data in the UK Met Office's UK and Regional Series are available from 1910 for each climate region as monthly, seasonal and yearly values. We used mean annual temperature and precipitation, as annual precipitation correlated with the precipitation for each season and the number of days with ≥1 mm rain in each season (Pearson's correlation: *r* ≥ 0.50, *p* ≤ 0.001), and mean annual temperature correlated highly with the yearly and seasonal maximum, minimum and mean temperatures (*r* ≥ 0.57, *p* ≤ 0.001; Figure S2; but we include, in the Supporting Information, additional analyses based on maximum and minimum annual temperatures). Although annual climatic data encompass months in which bumblebees are not developing, they provide an overview of the climatic conditions throughout each year, and they remove the need to make assumptions about the age of each bumblebee when collected, to infer when it had developed. However, we also provide analyses using climatic data from just the spring and summer months in the Supporting Information.

We focused on specimens collected between 1900 and 2000, which were represented by greater availability of data, with a recorded collection location and date, and with wings positioned in a manner in which venation could be assessed from a dorsal image (*Bombus hortorum* = 656; *B. lapidarius* = 818; *B. muscorum* = 511; and *B. pascuorum* = 1,351 individual specimens; Table S2). Additionally, we only considered queens collected later in the year. For example, for *B. hortorum*, we excluded queens collected before Julian day 150, as these likely represent queens that had developed in the year preceding collection and had overwintered (Julian day for *B. lapidarius* = 164; *B. muscorum* = 164; *B. pascuorum* = 159; Methods S1).

### 2.2 Wing landmarking

**FIGURE 1:**
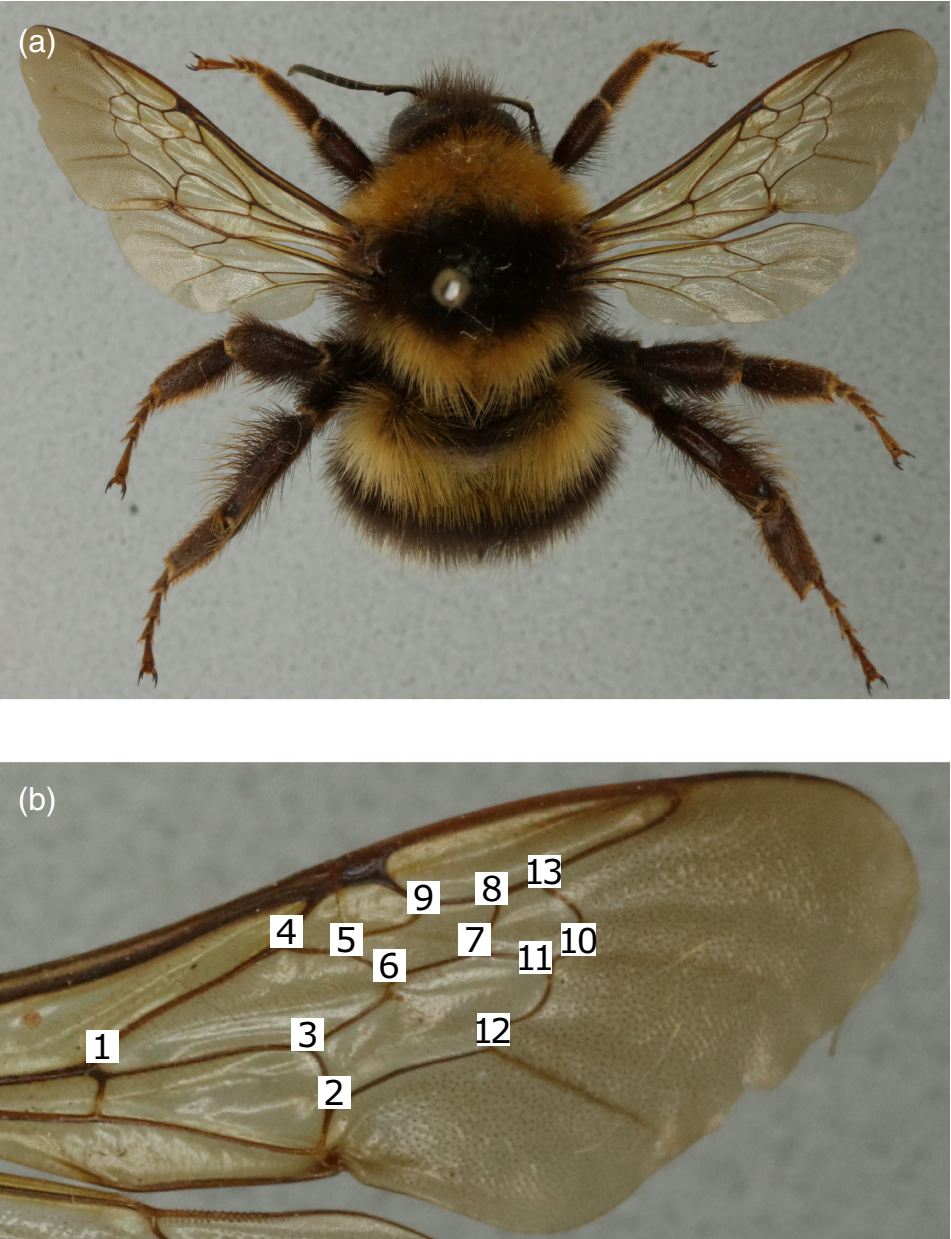
Digitised specimen (female *Bombus hortorum*). (a) Dorsal image of a pinned specimen. (b) Close‐up of the right forewing showing the homologous wing vein landmark numbers (white boxes used for visual purposes).

To capture forewing shape, we used the software tpsDig (v. 2.31; Rohlf, 2015) to manually plot 13 landmarks, which are homologous across bumblebees and have been used previously to investigate *FA* (Klingenberg et al., 2001; Figure 1). This resulted in two sets of 13 2D Cartesian coordinates per bumblebee (one set per forewing). Specimens were not re‐mounted or re‐positioned in any way before taking photographs to avoid damaging these irreplaceable and fragile specimens. Two people performed the landmarking, and to ensure consistent positioning of landmarks, we performed a repeatability analysis, where a subset of images (20 *B. hortorum* drones) were landmarked every 3 weeks over the landmarking period by both data collectors. For each specimen, there was no significant difference between wing shape measurements taken by the two data collectors over the landmarking period (linear mixed‐effects models; *F* = 0.203, *p* = 0.654; Table S3; Figure S3). Only images where all 13 landmarks were clearly visible were landmarked (Table S2; Methods S2).

### 2.3 Estimating wing shape asymmetry

Differences in wing scale, location and rotation were first removed (Kendall, 1977), by performing a Generalised Procrustes Alignment using the ‘geomorph’ package (Adams et al., 2020). Landmark configurations were scaled to the same size, and positions standardised by superimposing the centroids of all configurations on the origin. Variation in orientation was removed by rotating the landmarks around the centroid to fit each specimen to the ‘consensus’ (*viz*. the average positions of each landmark across all specimens; Tatsuta et al., 2018; Webster & Sheets, 2010). The coordinates from Procrustes superimposition formed the basis for all subsequent operations. Shape differences between the left and right forewings of an individual were estimated using Procrustes distance. This quantifies the mismatch in the landmarks between wings and is calculated as the square root of the sum of squared distances between the corresponding landmarks (greater distance represents larger *FA*).

To assess measurement error, all specimens were landmarked a second time, and the repeat measurements were subjected to a Procrustes analysis of variance (ANOVA; Side × Individual) for each species and sex (see Methods S3). We found no evidence of significant measurement error (Procrustes ANOVA; mean squares for measurement error <0.0002 relative to ≥0.00156 for Side × Individual across all species; Table S4; following Friedli et al., 2020).

### 2.4 Dealing with low‐quality images, angled wings and a skewed distribution of specimens

Tilt of the specimens relative to the position of the camera, image pixelation, differences in specimen illumination and specimens with warped wings can all cause error in measuring *FA* (Webster & Sheets, 2010). We therefore undertook two filtering steps: (a) we compared the wing shape of all specimens (of both their left and right forewings) to the mean wing shape across all specimens (using Procrustes distance) and removed all specimens that had a Procrustes distance above the upper quartile of this distribution; (b) we removed specimens with a wing‐angle differential (i.e. absolute difference between the left and right forewing angle) that was larger than the upper quartile plus the interquartile range (Methods S4; Table S5; Figure S4). This was because a large difference between the positioning (i.e. angles) of a specimen's wings (before Procrustes alignment) correlated significantly with higher wing shape *FA* in all species (all *r* > 0.18, *p* < 0.001; Figure S5). Therefore, by filtering out specimens with high *FA*, our findings and effect sizes can be considered conservative.

**FIGURE 2:**
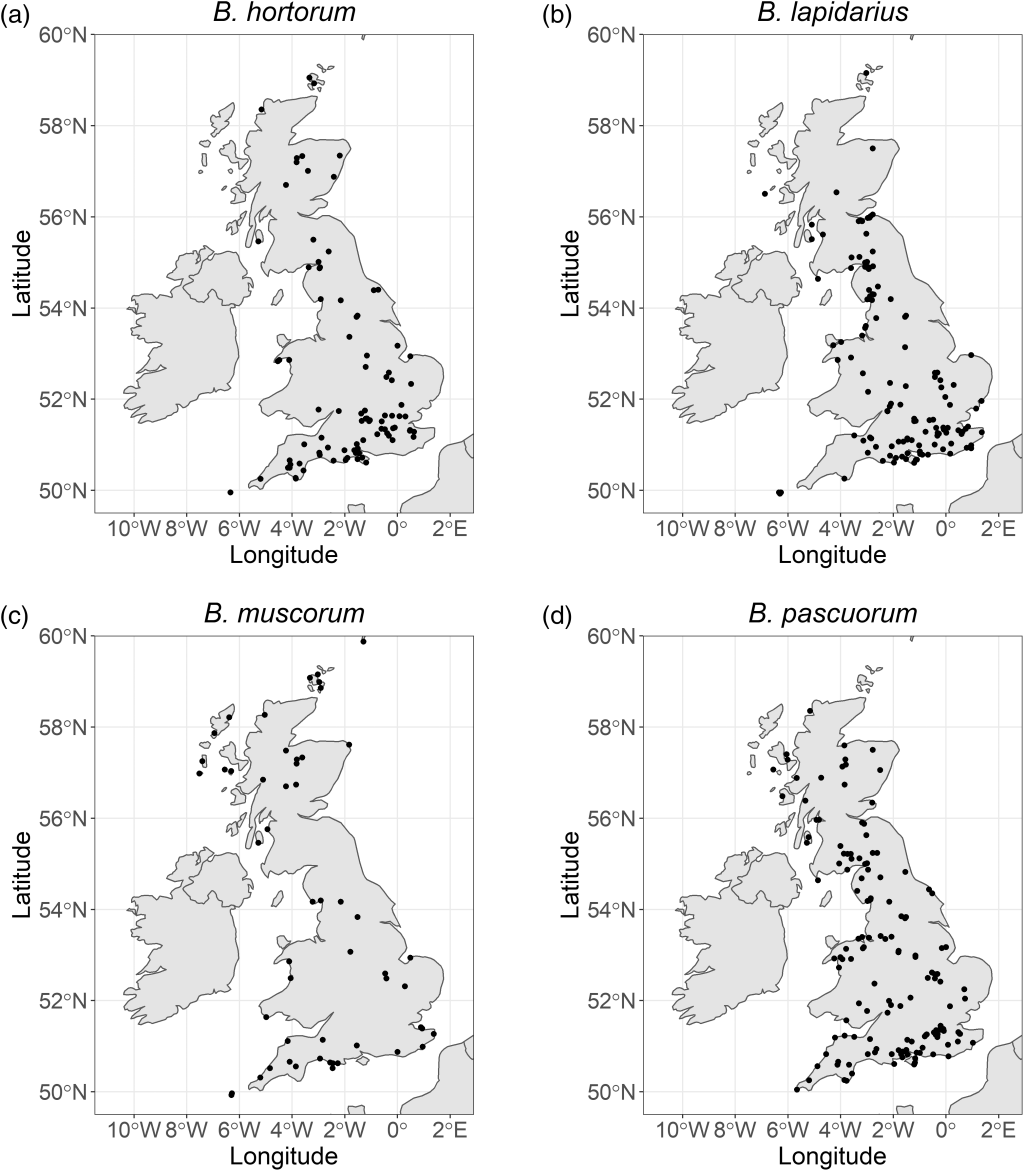
Distribution maps of *Bombus hortorum* (a), *B. lapidarius* (b), *B. muscorum* (c) and *B. pascuorum* (d) collected across Britain (sample size for *B. hortorum* = 133, *B. lapidarius* = 179, *B. muscorum* = 75, *B. pascuorum* = 203 specimens). R packages ‘rnaturalearth’ (South, 2017), ‘ggspatial’ (Dunnington & Thorne, 2020) and ‘ggplot2’ (Wickham et al., 2019) were used to produce this image.

We then implemented a third filtering step where we rarefied the data to ensure an even distribution of specimens across the 20th century. First, we reduced the dataset to contain a single randomly chosen specimen for each species (*n* = 4), month (*n* = 9), year (*n* = 93), climate region (*n* = 9) and caste (*n* = 3). Second, we limited each year to a maximum of 20 randomly selected specimens (as some years contained a disproportionately large number of specimens), to prevent heavily weighted years biasing trends over time, leaving 590 specimens for our analyses (Table S2; Figure 2).

### 2.5 Statistical analyses

We investigated how *FA* (dependent variable): (1) differed between the four bumblebee species when pooling all specimens across the century; (2a) responded between the two halves of the 20th century and (2b) whether the trend over the 20th century was nonlinear as a function of time; and (3) has responded nonlinearly with the combined effect of mean annual temperature and annual precipitation. For each of these three questions, we first fitted ‘maximal’ models containing all biologically relevant variables, and then simplified these maximal models by removing terms and comparing the reduced models using ANOVAs (with model variances compared against the *F* distribution); we additionally used these *F*‐tests to assess the significance of model terms and report them in Section 3. We additionally explored comparing reduced models using AIC values, and the results were qualitatively similar.

To examine how *FA* differed between the four species (1), our ‘maximal’ generalised additive mixed model (GAMM) predicted *FA* using species identity as a fixed effect; caste as a covariate (drones, workers and summer gynes [new queens]; controlling for the possibility of the different bumblebee castes differing in *FA*) and an interaction between species and caste; latitude and longitude combined in a smooth function, with a separate smooth for each species (controlling for the specimens being collected from different locations across Britain); and year (treating each year as a separate level within a factor) as a random effect (to control for the effects of *FA* changing over time). After model selection, the fitted GAMM included: caste; latitude and longitude, as a global smooth; and year as a random effect.

To examine how *FA* changed between the two halves of the century (2a), we divided the century into two periods (1900–1949 and 1950–2000), and modelled the interaction between century half and species identity using a generalised additive model (GAM). The maximal model additionally included caste as a covariate (and an interaction between species and caste), and latitude and longitude combined in a smooth function. The fitted GAM contained: caste; century half; and latitude and longitude, as a global smooth. To investigate the nonlinear relationship of time with *FA* (2b), we used a GAM and wrapped year in a smooth function. Year was scaled by subtracting 1899, to make the model intercept equal to the level of *FA* present at the beginning of the 20th century. Our maximal model gave each species their own intercept and smooth over the century. It additionally included caste as a covariate and an interaction between species and caste; and species‐level smooths for the combined effect of latitude and longitude. The fitted GAM included: caste; scaled year, as one global smooth; and latitude and longitude, as a global smooth.

Finally, for the combined effect of increasing mean annual temperature and annual precipitation (3), both variables were combined in a 2D tensor product smooth for each species in a GAMM. This maximal model additionally included species and castes as covariates (and their interaction); latitude and longitude combined in a smooth function, with a separate smooth for each species; and year (treating each year as a separate level within a factor) as a random effect. The fitted GAMM included: caste; mean annual temperature and precipitation combined in a global 2D tensor product smooth; latitude and longitude, as a global smooth; and year as a random effect.

All analyses were performed in R v.4.1.1 (R Core Team, 2021), with GAMs and GAMMs fitted using ‘mgcv’ package (Wood, 2012). Model diagnostic plots were checked for normally distributed residuals and homogeneity of variance. *FA* was log‐transformed to ensure model residuals were normally distributed. The fitted models were visualised using the ‘predict.gam’ function (Wood, 2012) and ‘ggplot2’ (Wickham et al., 2019).

## 3 RESULTS

### 3.1 Which bumblebee species show the highest levels of fluctuating asymmetry?

**FIGURE 3:**
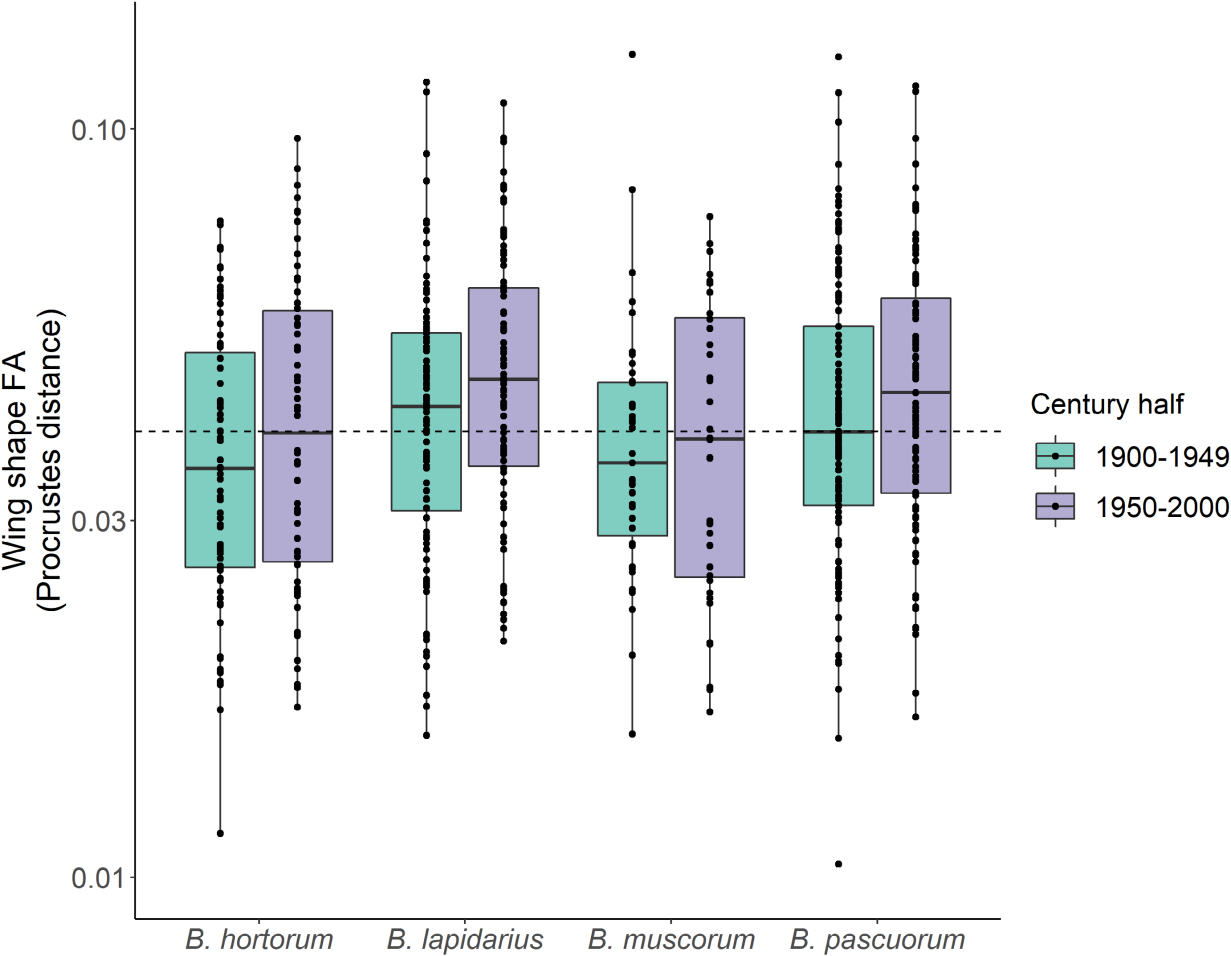
Bumblebee species likely differ in their baseline levels of wing shape fluctuating asymmetry (*FA*) and each show a consistently higher average *FA* in the second half of the century. The dashed line represents the median value of the species‐level medians. Individual points represent the raw *FA* values. Solid lines represent the median value per species; the lower and upper bounds of each box, the 25th and 75th percentiles respectively; and the lower and upper whiskers, the minimum and maximum values. *FA* values beyond the whiskers are deemed ‘outliers’ (i.e. 1.5 × the interquartile range outside of the box). Note that the *y*‐axis is on a log scale.

Wing shape *FA* indicated differences between the four bumblebee species (*F*‐test: model degrees of freedom [DF] for GAMMs with and without ‘species’ intercepts = 46.5 & 44.2, respectively, *F* = 2.57, *p* = 0.067; Tables S6 and S7; Figure 3; Figure S6), with *B. pascuorum* and *B. lapidarius* (mean *FA* = 0.0462 & 0.0465 ± standard error 0.00144 & 0.00145 respectively) having significantly higher *FA* than *B. hortorum* (*B. hortorum* mean *FA*: 0.0411 ± 0.00161; *B. muscorum* mean *FA*: 0.0408 ± 0.00216; GAMM: model DF = 46.5, *t* = 2.39, *p* = 0.0173; *t* = 1.98, *p* = 0.0487, respectively; adjusted *R* ^2^ = 15.7%).

### 3.2 Change in fluctuating asymmetry over the 20th century

When investigating how *FA* changed between the first half of the century (1900–1949) and the second (1950–2000; Figure 3), there was a significant increase in *FA* (*F*‐test: model DF for GAMs with and without ‘century half’ intercepts = 16.9 & 16.6, respectively, *F* = 7.40, *p* = 0.034; Tables S8–S11) that was consistent across all four bumblebee species (i.e. no significant interaction between century half and species identity; *F*‐test: model DF for GAMs with and without this interaction = 22.3 and 19.2, respectively, *F* = 0.541, *p* = 0.7; Table S9). This represented a mean increase of 9.09% (±2.32%) in *FA* for the four bumblebee species between the first and second halves of the 20th century.

**FIGURE 4:**
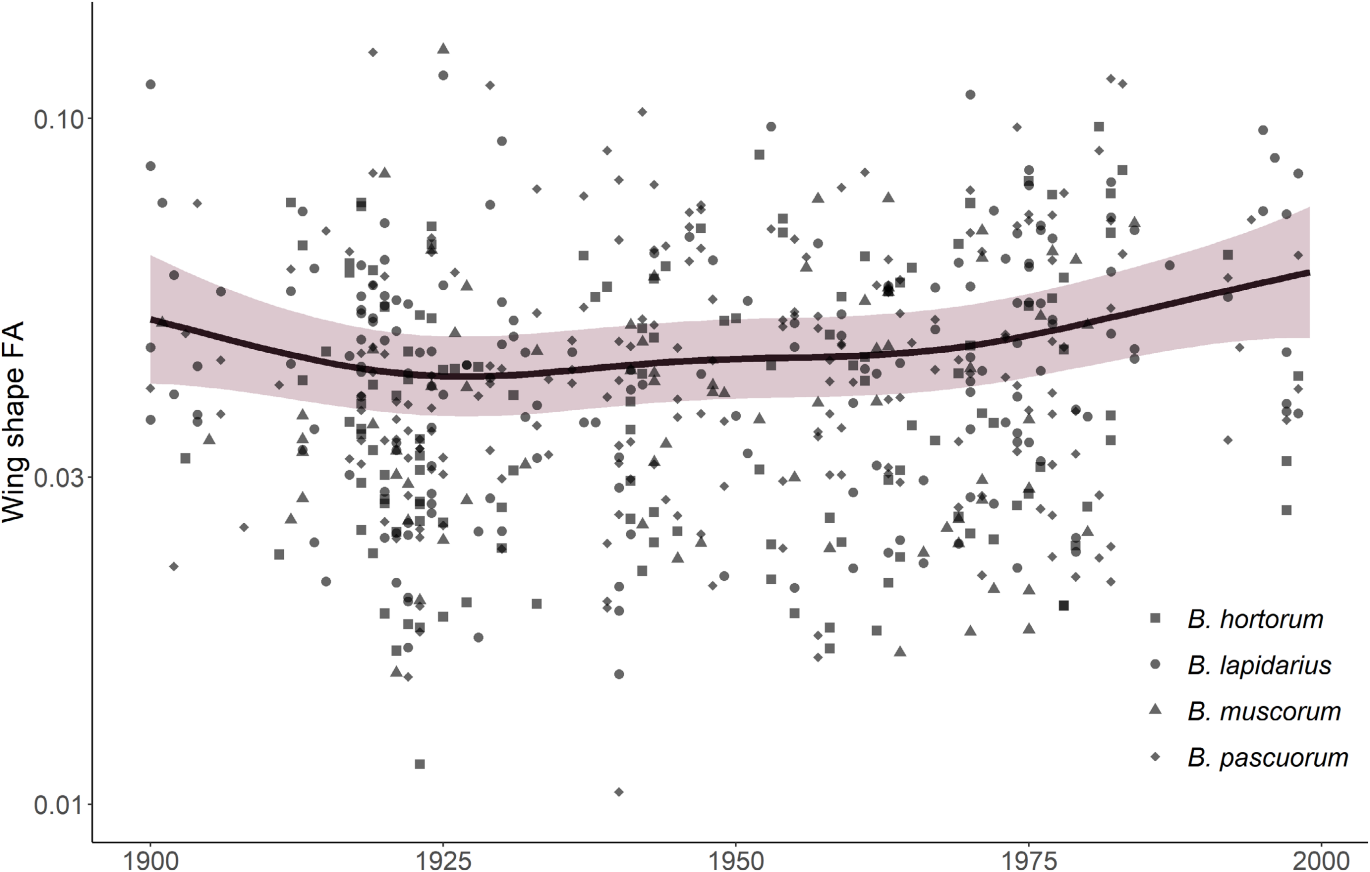
Nonlinear trend in wing shape fluctuating asymmetry (*FA*) over the 20th century. Points represent the raw *FA* data for all species, with different shapes for the different bumblebee species. The solid black line shows the generalised additive model (GAM) prediction for all species (based on a worker bumblebee at mean latitude and longitude, as the model prediction accounts for variation in *FA* across bumblebee castes and sample collection locations). Shading shows 2 × standard error of the GAM estimates. Note that the y‐axis is on a log scale. Sample size of *B. hortorum* = 133, *B. lapidarius* = 179, *B. muscorum* = 75 and *B. pascuorum* = 203 specimens.

We next analysed the continuous, nonlinear change in *FA* over the 20th century, finding that species did not differ significantly in their trends (smooths estimates with time), again indicating that the four species are responding in a similar manner (*F*‐test: model DF for GAMs with and without species‐specific smooths = 24.7 and 22.1, *F* = 1.43, *p* = 0.239; Tables S12–S15). Overall, bumblebees exhibited a continual increase in *FA* after *c*. 1925 (GAM: effective degrees of freedom [EDF] = 3.65, *F* = 4.03, *p* = 0.00163; adjusted *R* ^2^ = 11.0%; Table S14; Figure 4), with mean *FA* increasing 8.81% from 0.0489 (±0.0112) in 1925 to 0.0532 (±0.00878) in 1998.

### 3.3 Changes in fluctuating asymmetry with environmental variables

**FIGURE 5:**
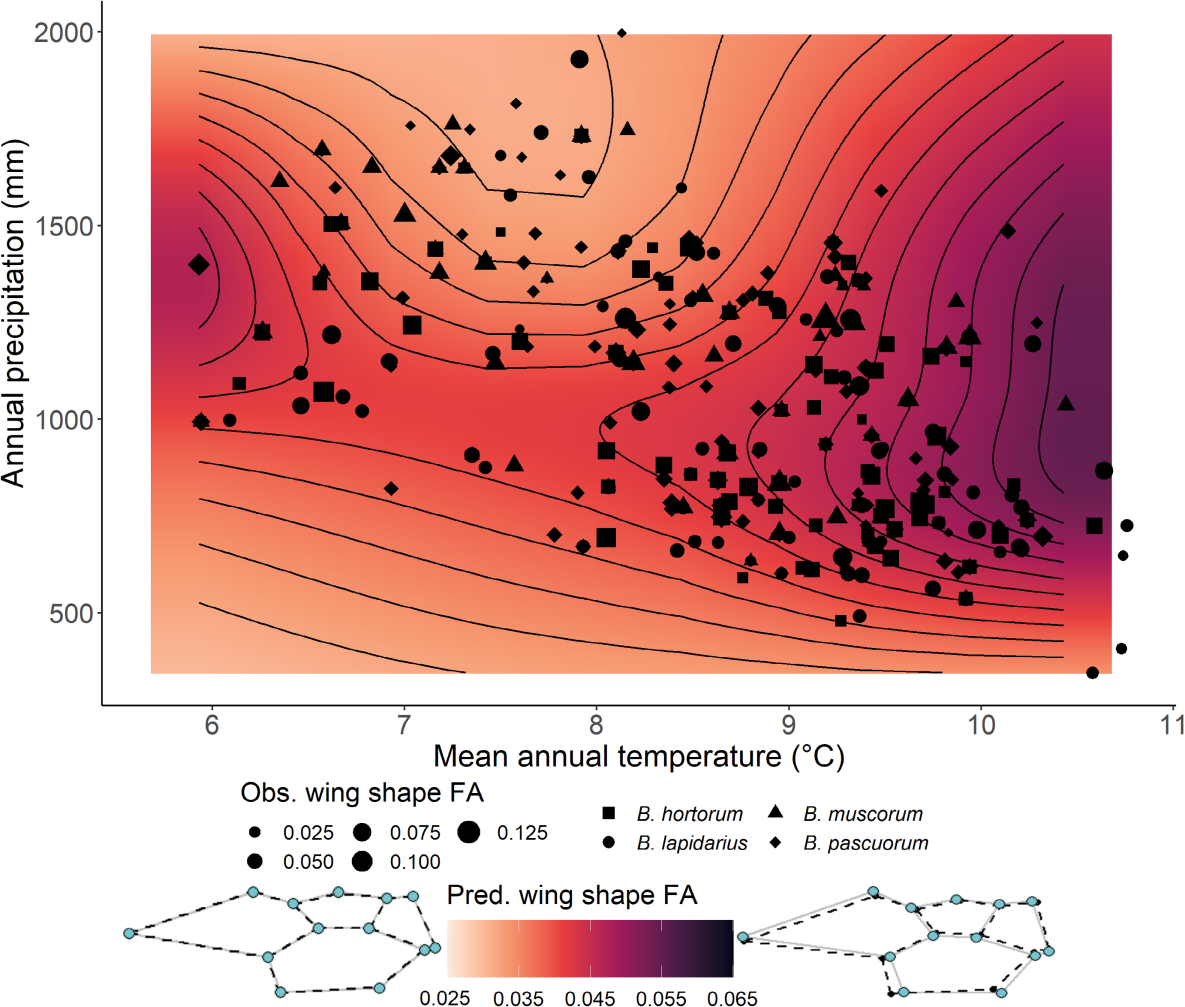
Bumblebee wing shape fluctuating asymmetry (*FA*) changes nonlinearly with the combined effect of mean annual temperature and total annual precipitation. Heatmap colours represent GAMM predictions of *FA* for workers sampled at mean latitude and longitude, as the GAMM used to predict *FA* accounted for variation across bumblebee castes and sample collection locations. Darker colours represent higher predicted (‘Pred.’) *FA*. Points indicate the raw (‘Obs.’) *FA* values, with the size indicating the magnitude and different shapes representing the four species. To visualise what wings look like with relatively low (Procrustes distance = 0.010) and high (0.065) *FA*, two sets of wing outlines are placed either side of the scale bar, representing the lower and upper predicted asymmetry ranges respectively. Grey lines with blue points represent right forewings, and black dashed lines with black points represent left forewings (superimposed onto the right wings using Procrustes alignment). Wing outlines were created using a modified version of the ‘plotRefToTarget’ function from the ‘geomorph’ package (Adams et al., 2020).

When investigating how *FA* changes in response to the combined effect of mean annual temperature and annual precipitation, we found the trends (smooth estimates) did not vary between species (*F*‐test: model DF for GAMMs with and without species‐specific smooths = 60.8 and 48.0, *F* = 1.09, *p* = 0.354; Tables S16–S19), indicating the four species responded similarly. Indeed, we found a significant interaction between the two variables for bumblebees as a whole (GAMM: EDF = 8.99, *F* = 2.33, *p* = 0.00806; adjusted *R* ^2^ = 17.7%; Table S19; Figure 5), with higher *FA* under warmer years with intermediate rainfall (>9°C & 750–1,250 mm/year). Results for analyses investigating how *FA* is associated with seasonal (spring and summer) climate variables, and annual maximum and minimum temperature, can be found in Figures S7–S10 and Tables S20–S27.

## 4 DISCUSSION

By measuring differences in the wing morphology of museum specimens, we investigated levels of fluctuating asymmetry (*FA*) over the 20th century as a potential proxy of stress experienced by bumblebee populations. We found that our four studied species likely vary in their baseline stress (*FA*), with *B. pascuorum* and *B. lapidarius* showing higher levels. All species, however, showed a similar temporal response, as the trend in *FA* appeared to show a gradual increase after *c*. 1925 to the end of the century. Moreover, high *FA* was associated with warmer and wetter years (mean annual temperature > 9°C; annual precipitation: 750–1,250 mm), with all species again showing a consistent response. Overall, these results could suggest bumblebees experienced increasing stress as the century progressed and that aspects of climate change could have contributed to this trend.

### 4.1 Bumblebee species likely differ in their baseline levels of fluctuating asymmetry

Our results indicate that the degree of baseline *FA* can vary even between relatively closely related species, with lower levels of *FA* in *B. hortorum* and *B. muscorum* relative to *B. pascuorum* and *B. lapidarius*. Intriguingly, the lower levels of *FA* in *B. muscorum* are seemingly at odds with its status as a vulnerable species (Rasmont et al., 2015) and a relative ‘loser’ from environmental change, as evidenced by large range contractions in Britain since the 1960s (Casey et al., 2015). However, given the rarity of *B. muscorum* in the latter half of the century, the low levels of *FA* could be explained by *B. muscorum* being primarily confined to its favoured habitat (mainly coastal regions, with tall, flower‐rich grasslands; Edwards, 2012). Consequently, low *FA* may be an artefact of natural history collectors being unable to sample specimens from locations where *B. muscorum* would be rarer and possibly subject to greater environmental stressors (e.g. Adamski & Witkowski, 2002). Using *FA* as a possible signature of stress may therefore be more useful for detecting early‐warning signs for generalist species experiencing a wider range of conditions (i.e. a larger geographic distribution) than for more specialist species with narrower habitat requirements. The higher baseline levels of *FA* in *B. pascuorum* and *B. lapidarius* are also interesting, as these are the two more common species and both have recently expanded their ranges northwards (Casey et al., 2015). Higher *FA* could therefore be a potential signature of stress when colonising new environmental and climatic conditions (Krause et al., 2016; Liebl & Martin, 2013). Furthermore, this result suggests that, although *FA* may be an indicator of stress in populations (Beasley et al., 2013; Clarke, 1995; Leary & Allendorf, 1989; Parsons, 1992; Van Dongen, 2006), high *FA* levels in a population may not always equate to negative population outcomes (reviewed in Clarke, 1995), and, indeed, not all studies have detected a link between high *FA* and stress (e.g. Bjorksten et al., 2001; Floate & Fox, 2000; Servia et al., 2004; Ward et al., 1998). Previously, this has been suggested to be due to studies having small sample sizes (Babbitt et al., 2006), large measurement error (Palmer, 1994) or measuring traits less susceptible to *FA* (Arundell et al., 2019; Lazić et al., 2013). However, the lack of association between *FA* and fitness here may be as result of the most resilient individuals being able to bear higher stress levels (similar to Zahavi's (1975) handicap principle).

### 4.2 Signatures of higher fluctuating asymmetry in the latter half of the 20th century

When investigating trends over the century, *FA* showed a relative increase for all four species after *c*. 1925, with *FA* levels at the end of the century higher than pre‐1925 levels. Although drivers of stress are multifactorial and complex (Zaragoza‐Trello et al., 2021), this trend of increasing *FA* coincides with large changes in agricultural practices, driven by government policies, that occurred in Britain following the First World War and during and after World War II, including expanding arable land and increasing use of pesticides and herbicides (Ollerton et al., 2014; Robinson & Sutherland, 2002). Such practices have individually been shown to increase *FA* in laboratory and field experiments in a wide range of invertebrates (Abaga et al., 2011; Chang et al., 2007; Costa & Nomura, 2016; Friedli et al., 2020; Mpho et al., 2001) and vertebrates (Coda et al., 2016). Alternatively, other global change drivers, such as emergent diseases (Fürst et al., 2014; Yordanova et al., 2022), spread of invasive species (Meeus et al., 2011) and climate change (Forister et al., 2018; Halsch et al., 2021; Kerr et al., 2015), may also explain increasing *FA* over the latter part of the century. Additionally, the past century has seen possible increased competition between managed honeybees and wild bumblebee species (Thomson, 2016). Although to our knowledge no studies have assessed the link between bumblebee interspecific competition and *FA*, competition between larvae is associated with higher levels of *FA* in butterflies (e.g. *Parage aegeria*; Gibbs & Breuker, 2006). Indeed, a growing body of evidence supports the idea that environmental stressors can impact individuals not just as adults but from the start of their life cycle, that is, during larval development (Pellegroms et al., 2009; Smith et al., 2020). But regardless of the exact mechanism, quantifying *FA* has potentially given insights into historic stress, and specifically provided a more temporally accurate assessment of when this may have been experienced in wild bumblebee populations.

Despite the consistent trend across species over time, we note that unexplained variance in *FA* remains. This could be due to differences in local habitat or microclimate (Henriques & Cornelissen, 2019; Kark, 2001; Schmeller et al., 2011) or other stressful conditions, which could interact in non‐additive ways (Zaragoza‐Trello et al., 2021). Alternatively, variance in *FA* could also be influenced by the positioning of wings on museum specimens. However, as *FA* was not higher in older specimens, we can be confident that the increase in *FA* over time is not a result of damage to specimens within individual collections. Ideally, *FA* would be measured on wings that have been detached from the specimen and slide mounted. However, as we are dealing with irreplaceable biological artefacts, a challenge for future studies would be to develop high‐throughput scanning methods to more accurately measure *FA* from wings of pinned museum specimens (e.g. Perrard et al., 2012; Plum & Labonte, 2021).

### 4.3 Warm and wet years are associated with high fluctuating asymmetry

*FA* was consistently higher in warmer and wetter years. Importantly, this finding seems to support other studies finding a strong signal of climate in driving insect population trends (Forister et al., 2018; Halsch et al., 2021; Kerr et al., 2015; Román‐Palacios & Wiens, 2020; but see Guzman et al., 2021). It is also consistent with most bumblebee populations being better adapted to colder conditions, with different populations—regardless of whether they are from warm or cold environments—having similar tolerances to high temperatures (Martinet et al., 2021; Pimsler et al., 2020). Moreover, temperature stress has been linked to increased *FA* across a range of invertebrate species (Gerard et al., 2018; Imasheva et al., 1997; Nishizaki et al., 2015; Vishalakshi & Singh, 2008). Additionally, warmer, wetter years could promote pathogen spread and infection (Lafferty, 2009; Neidel et al., 2017), which are also associated with increased levels of *FA* (Arundell et al., 2019). Given that mean annual temperature for many regions will likely increase under climate change, our findings could reveal an early‐warning sign, with some species potentially being unable to sustain continued increases in *FA*. Alternatively, these climatic conditions could correlate with increased nutritional stress, as declines in floral abundance, and consequent loss of insect pollinators, have been reported with climate extremes (Høye et al., 2013; Iserbyt & Rasmont, 2012; Thomson, 2016). Expanding this analysis, and network of museums, to include more bumblebee species and regions representing the southern and northern limits of species' ranges that may experience more severe climate change (such as the Mediterranean and Arctic regions in Europe) is therefore a research priority, to understand the extent of impact that natural populations will face in the future.

## 5 CONCLUSIONS

By leveraging morphological data in museum specimens, this study has, for the first time, tracked *FA* over an extended period and at a temporal resolution that potentially allows more accurate assessment of when during the century bumblebee populations may have experienced stressors. By using wing shape *FA* (rather than other measures of a population's response, such as geographic distribution), we avoided potential lag effects of stressors that often obscure how populations are responding at a given time point. Although the relationship between *FA* and stress is not solely determined by environmental stressors, our results have potentially revealed periods of historical signatures of developmental stress worthy of further investigation. Moreover, across our studied species, increases in *FA* over the 20th century were found in warmer, wetter years. Moving forward, measuring *FA* in contemporary specimens, and especially relating it to land‐use and climate change hotspots, may provide a mechanistic insight into the causes of stress and provide early‐warning indicators to inform safeguarding strategies to protect beneficial insects. Ultimately, our study highlights the crucial role long‐term collections of museum specimens can play in understanding past and future pollinator responses to environmental change.

## AUTHOR CONTRIBUTIONS

Andres N. Arce, Ian Barnes, Selina Brace, Jeff Ollerton and Richard J. Gill conceived the project, with additional input from David Notton. Andres N. Arce, Aoife Cantwell‐Jones, Michael Tansley & Richard J. Gill designed the study. Andres N. Arce oversaw digitisation of the specimens and collated the metadata, with direct contributions from Phaedra Kokkini, Victoria E. Mullin, David Notton, James Hogan, Tony Hunter, Steve Judd and Ashleigh Whiffin. Curatorial support was further provided by Vladimir Blagoderov, Gavin Broad and Steve Judd. Phaedra Kokkini and Laurence Livermore were involved in digitisation of the NHM London collection. Marcus W. Rhodes digitised and extracted metadata for the Oxford specimens. Emma Eatough, Marcus W. Rhodes, Xueni Bian, Aoife Cantwell‐Jones, Michael Tansley, Andres N. Arce and Richard J. Gill landmarked the bumblebee wings. Aoife Cantwell‐Jones and Andres N. Arce performed the data analyses, with input from Mahika K. Dixit, William D. Pearse and Richard J. Gill. Aoife Cantwell‐Jones, Andres N. Arce and Richard J. Gill wrote the manuscript, with input from all coauthors. All authors gave final approval for publication.

## FUNDING INFORMATION

The work was funded by NERC grants: NE/P012574/1 and NE/P012914/1 awarded to Richard J. Gill and Ian Barnes, which supported Andres N. Arce, Jeff Ollerton, Selina Brace and Victoria E. Mullin. Aoife Cantwell‐Jones and Mahika K. Dixit are supported by the NERC Science and Solutions for a Changing Planet (SSCP) DTP program (NE/S007415/1).

## CONFLICT OF INTEREST

The authors declare no conflict of interest.

## Supporting information


Appendix S1

## Data Availability

Data available from the Environmental Information Data Centre (EIDC) repository https://doi.org/10.5285/2696535e‐564a‐4c6a‐877e‐515996fa97a1 (Arce et al., 2022).
