# Supplementary material for: Signatures of increasing environmental stress in bumblebee wings over the past century: Insights from museum specimens
Source: J Anim Ecol. 2022 Aug 17;92(2):297–309. doi: 10.1111/1365-2656.13788 (PMC10086799; doi:10.1111/1365-2656.13788)
Supplement: Supplementary file 1 — Appendix S1 [file JANE-92-297-s001.docx]

**Signatures of increasing environmental stress in bumblebee wings over the past century: Insights from museum specimens**

Arce & Cantwell-Jones et al.

**SUPPLEMENTARY METHODS**

Supplementary Methods 1: **Assigning queen cohorts**

We assigned every individual per species to one of three phenophases (an early, middle and late cohort), to identify and exclude any queen-sized individuals that had emerged from hibernation in the spring (the early cohort), as these individuals will have developed in the previous year. We adapted the method of Duchenne et al. (2020) and fitted a generalised additive model that incorporated the latitude and longitude for each species, i.e. statistically correcting for the effect of location. We then assigned membership to each cohort using a Gaussian mixture model to cluster the individual samples into three distinct groups based on their observation date using the “*densityMclust*” function in *mclust”* (Scrucca et al., 2016). Including the early queens in our analysis would have required assigning them to the previous year. Here we decided not to do this, given we do not know how overwintering may filter out low-quality individuals leading to either inflated or deflated levels of fluctuating asymmetry.

Supplementary Methods 2: **Identifying and correcting for specimens with upside down wings.**

First, specimens were categorised as either having wings arranged perpendicular to their body (horizontal) or on their abdomen (vertical), by subtracting the y-coordinate for landmark (LM) 1 from LM 10 (Fig. 1). This difference formed a bimodal distribution; values smaller than the trough were classified as “horizontal”; those larger, as “vertical”; the exact cut-off point was determined by manually checking the original images. Then, flipped wings were identified by subtracting LM 2 from 4. For horizontal and vertical wings, if the differences in the y- and x-coordinates, respectively, were negative, the wing was “flipped”. Flipped wings then had their y-coordinates multiplied by -1, to transform the coordinates into the same plane as “non-flipped” wings.

Supplementary Methods 3: **Assessing measurement error and directional asymmetry.**

Deviations from bilateral symmetry can sometimes be genetically determined, for example directional asymmetry, where the degree of asymmetry is consistently biased in one direction (like the human heart), and anti-symmetry, when one side is always larger than the other, but the side varies across individuals (such as male fiddler crab claws; *Uca lactea*). There is debate about the extent to which directional asymmetry and anti-symmetry are adaptive forms of asymmetry or whether they can also represent an individual’s ability to buffer developmental stability (Kark, 2001). Directional asymmetry was tested for using a separate two-way Procrustes analysis of variance (ANOVA; Side × Individual) for each species and caste (Graham et al., 2010). If the term “Side” (viz. left or right forewing) is significant, there is significant directional asymmetry. The significance of the “Side × Individual” term represents significant *FA*. As *FA* is estimated relative to the measurement error, a significant term suggests low measurement error (as in Friedli et al., 2020). Our results are presented in Supplementary Table 4.

Supplementary Methods 4: **Filtering low-quality specimens.**

We removed any specimens that possessed a wing shape showing a large difference to the mean wing shape from the analysis (viz. with a Procrustes distance to the mean shape above the upper quartile). Additionally, specimens with a wing-angle differential (i.e. the absolute difference between the left forewing angle and right forewing angle) larger than the interquartile range above the upper quartile were removed. These two quality-control thresholds were set by trying to maximise the number of specimens remaining while removing as many “low-quality” images as possible (Fig. S4; Table S2 & S4). We assessed thresholds of 0, 1 and 1.5 times the interquartile range of the distributions for both distance to the mean wing shape and wing-angle differential, and all possible combinations (Fig. S4). There were no significant differences in the variances of the wing shape *FA* values between the most stringent cut-off tested (both thresholds were 0; Fig. S4I), and the cut-offs we chose (wing-shape threshold = 0, wing-angle threshold = 1; Fig. S4G; *F-*tests; all species *F* <1.01, *p* >0.76; Table S5).

**SUPPLEMENTARY TABLES**

| Supplementary Table 1: **Empirical studies cited in this manuscript that find a significant relationship between fluctuating asymmetry (*FA*) and a given stressor.** Note the purpose of this table is not to review all *FA* literature, but to highlight a range of organisms for which fluctuating asymmetry has been found to be a proxy for stress. Full citations for each study can be found in the main manuscript. | | |
| --- | --- | --- |
| Study | Stressor tested | Taxa studied |
| Abaga et al., 2011 | Insecticide residues | *Apis mellifera* (honeybee) |
| Adamski & Witkowski, 2002 | Population extinction | *Parnassius apollo frankenbergeri* (Apollo butterfly) |
| Al-Shami et al., 2011 | Water quality & metal pollution | *Chironomus* spp. (non-biting aquatic midges) |
| Arundell et al., 2019 | Parasitism | *Gammarus duebeni* & *G. zaddachi* (Gammarid crustacean) |
| Bonn et al., 1996 | Ectoparasitic mites | *Coenagrion* *puella* (damselfly) |
| Chang et al., 2007 | Insecticide | *Ceriagrion* spp. (damselfly) |
| Chirichella et al. 2020 | Population density, forage quality, length of snow cover | *Rupicapra* *rupicapra* (Alpine chamois) |
| Coda et al., 2016 | Farming practice | *Akodon* *azarae* (Pampean grassland mouse) |
| Costa & Nomura, 2016 | Herbicide | *Physalaemus* *cuvieri* (tadpoles) |
| Friedli et al., 2020 | Insecticides | *Apis* *mellifera* (honeybee) |
| Gerard et al., 2018 | Nutritional stress, temperature stress | *Bombus* *terrestris* (bumblebee) |
| Gibbs & Breuker, 2006 | Larval-rearing density | *Pararge* *aegeria* (speckled wood butterfly) |
| Graham, Roe & West, 1993 | Lead and benzene exposure | *Drosophila* *melanogaster* (fruitfly) |
| Groenendijk, Zeinstra & Postma, 1998 | Metal contamination | *Chironomus* *riparius* (non-biting aquatic midges) |
| Henriques & Cornelissen, 2019 | Altitude | Butterfly community |
| Hosken, Blanckenhorn & Ward, 2000 | Heat stress | *Scathophaga* *stercoraria* (yellow dung flies) |
| Imasheva et al., 1997 | Heat stress | *Drosophila* *melanogaster* & *D. buzzatii* |
| Kark, 2001 | Distance to range edge (though note use of directional asymmetry) | *Alectoris* *chukar* (chukar partridge) |
| Klingenberg et al., 2001 | Elevated CO_2_ | *Bombus* *empatiens* (bumblebee) |
| Lazić et al., 2013 | Urbanisation | *Podarcis* *muralis* (common wall lizard) |
| Leonard et al., 2018 | Urbanisation | *Apis* *mellifera* (honeybee) |
| Møller, Erritzøe & Van Dongen, 2018 | Summer temperature | Birds |
| Mpho, Holloway & Callaghan, 2001 | Insecticide exposure | *Culex* *quinquefasciatus* (mosquitoes) |
| Nishizaki, Barron & Carew, 2015 | Heat stress | Mussels |
| Schmeller et al. 2011 | Response to conservation efforts | *Parnassius* *apollo* (Apollo butterfly) |
| Simbula et al., 2021 | Farming practice | *Podarcis* *siculus* (wall lizard) |
| Stringwell et al., 2014 | Survival after release from a hatchery | *Salmo* *salar* (Atlantic salmon) |
| Talloen et al., 2004 | Nutritional stress | *Pararge* *aegeria* (speckled wood butterfly) |
| Tocts, Johnson & Carter, 2016 | Survival in wild populations | *Brachyistius* *frenatus* (kelp perch) |
| Trotta et al., 2005 | Heat stress | *Drosophila* *melanogaster* (fruit fly) |

| Supplementary Table 2: **Sample size for each bumblebee species at each stage of data filtering.** | | | | |
| --- | --- | --- | --- | --- |
|  | *B. hortorum* | *B. muscorum* | *B. lapidarius* | *B. pascuorum* |
| Images landmarked | 656 | 511 | 818 | 1,351 |
| After low-quality images removed | 373 | 292 | 484 | 787 |
| After rarefaction & removing spring gynes | 133 | 75 | 179 | 203 |

| Supplementary Table 3: **Measurement of shape fluctuating asymmetry (*FA*) over the landmarking period.** To ensure consistent landmarking, 20 images (*B. hortorum* drones) were landmarked every three weeks over the landmarking period. Linear mixed-effects models were used to ensure no significant differences in the estimated wing shape FA for each specimen (the random effect) occurred over the landmarking period or due to the person who performed the landmarking. An analysis of variance was subsequently performed on the mixed-effects model. Bolded values are statistically significant (*p* <0.05). | | | | |
| --- | --- | --- | --- | --- |
| Coefficient | Estimate | Standard error | *T* | *P* |
| Intercept (Landmarker: Aoife, date: 19/12/19) | -2.71 | 0.162 | -16.8 | **<0.001** |
| Landmarker: Michael | 0.0146 | 0.0210 | 0.695 | 0.490 |
| Date: 05/01/20 | -0.0160 | 0.0205 | -0.779 | 0.439 |
| Date: 28/01/20 | -0.0178 | 0.0198 | -0.899 | 0.372 |
| Michael × 05/01/20 | -0.0636 | 0.0294 | -0.216 | 0.830 |
| Michael × 28/01/20 | -0.0216 | 0.0291 | -0.742 | 0.461 |
|  |  |  |  |  |
|  | Variance | Standard deviation |  |  |
| Random intercept: Specimens | 0.391 | 0.626 |  |  |
| Residual variance | 0.00280 | 0.0529 |  |  |
|  |  |  |  |  |
| Analysis of variance on the mixed-effects model | | | | |
| Coefficient | Sum of squares | Mean squares | *F* | *P* |
| Landmarker | <0.001 | <0.001 | 0.203 | 0.654 |
| Date landmarked | 0.0111 | 0.00557 | 1.99 | 0.145 |
| Landmarker × Date | 0.00163 | <0.001 | 0.291 | 0.748 |

| Supplementary Table 4: **Calculated wing shape fluctuating asymmetry for *Bombus hortorum, B. lapidarius,* *B. muscorum* and *B. pascuorum* sexes using Procrustes analysis of variance.** The term “Individual” refers to differences between specimens. Significant “Side” coefficient indicates significant directional asymmetry. “Individual × Side” is estimated relative to the “Measurement error”; a significant “Individual × Side” term indicates both a small measurement error and significant fluctuating asymmetry. Wing shape asymmetry was estimated using Procrustes distance. Values in bold are statistically significant (*p* <0.05). | | | | | |
| --- | --- | --- | --- | --- | --- |
| *Bombus hortorum* | | | | | |
| Females |  | Mean square (MS) | DF | *F* | *P* |
|  | Individual | 0.00678 | 232 | 4.35 | 0.991 |
|  | Side | 0.00798 | 1 | 5.12 | **0.003** |
|  | Individual × Side | 0.00156 | 232 | 0.751 | **0.002** |
|  | Measurement error | <0.0001 | 450 |  |  |
|  |  |  |  |  |  |
| Males |  | MS | DF | *F* | *P* |
|  | Individual | 0.0825 | 146 | 4.61 | 0.979 |
|  | Side | 0.00840 | 1 | 4.70 | **0.003** |
|  | Individual × Side | 0.00179 | 146 | 0.670 | **0.002** |
|  | Measurement error | <0.0001 | 274 |  |  |
|  |  |  |  |  |  |
| *Bombus muscorum* | | | | | |
| Females |  | MS | DF | *F* | *P* |
|  | Individual | 0.00904 | 215 | 4.16 | 0.993 |
|  | Side | 0.00232 | 1 | 1.07 | 0.335 |
|  | Individual × Side | 0.00217 | 215 | 0.746 | **0.002** |
|  | Measurement error | 0.000105 | 414 |  |  |
|  |  |  |  |  |  |
| Males |  | MS | DF | *F* | *P* |
|  | Individual | 0.00966 | 67 | 4.84 | 0.882 |
|  | Side | 0.00403 | 1 | 2.02 | 0.089 |
|  | Individual × Side | 0.00199 | 67 | 0.668 | **0.002** |
|  | Measurement error | <0.0001 | 136 |  |  |
|  |  |  |  |  |  |
| *Bombus lapidarius* | | | | | |
| Females |  | MS | DF | *F* | *P* |
|  | Individual | 0.0106 | 314 | 4.83 | 0.917 |
|  | Side | 0.0151 | 1 | 6.88 | **0.001** |
|  | Individual × Side | 0.00219 | 314 | 0.642 | **0.002** |
|  | Measurement error | 0.000193 | 584 |  |  |
|  |  |  |  |  |  |
| Males |  | MS | DF | *F* | *P* |
|  | Individual | 0.00875 | 161 | 4.26 | 0.955 |
|  | Side | 0.00496 | 1 | 2.41 | **0.036** |
|  | Individual × Side | 0.00205 | 161 | 0.687 | **0.002** |
|  | Measurement error | 0.000153 | 304 |  |  |
|  |  |  |  |  |  |
| *Bombus pascuorum* | | | | | |
| Females |  | MS | DF | *F* | *P* |
|  | Individual | 0.0106 | 495 | 5.30 | 0.996 |
|  | Side | 0.0111 | 1 | 5.57 | **0.002** |
|  | Individual × Side | 0.00200 | 495 | 0.618 | **0.002** |
|  | Measurement error | 0.000101 | 956 |  |  |
|  |  |  |  |  |  |
| Males |  | MS | DF | *F* | *P* |
|  | Individual | 0.00724 | 266 | 3.45 | 1.00 |
|  | Side | 0.00547 | 1 | 2.61 | **0.033** |
|  | Individual × Side | 0.00210 | 266 | 0.861 | **0.002** |
|  | Measurement error | <0.0001 | 510 |  |  |
|  |  |  |  |  |  |
|  |  |  |  |  |  |

| Supplementary Table 5: **Results of *F-*tests comparing the variances of wing shape fluctuating asymmetry values for specimens included under the specimen quality cut-offs used and the most stringent cut-offs** (“chosen cut-offs” – a wing angle differential threshold of 1 times the interquartile range above the upper quartile and a wing shape within the interquartile range above the upper quartile of the mean wing shape; and “stringent cut-offs” – wing angle differential threshold of the interquartile range above the upper quartile and a wing shape within the interquartile range above the upper quartile of the mean wing shape; respectively). | | | | |
| --- | --- | --- | --- | --- |
| Species | Specimens with chosen cut-offs (*N*) | Specimens under most stringent cut-offs (*N*) | *F*-value | *p-value* |
| *B. hortorum* | 373 | 306 | 1.01 | 0.941 |
| *B. pascuorum* | 788 | 642 | 1.00 | 0.999 |
| *B. lapidarius* | 484 | 394 | 0.992 | 0.933 |
| *B. muscorum* | 292 | 237 | 0.963 | 0.764 |

| Supplementary Table 6: **Generalised additive mixed model estimates to compare fluctuating asymmetry between the four bumblebee species.** The response variable was (logged) wing shape fluctuating asymmetry. R^2^ = 0.157, *N* = 587. EDF = effective degrees of freedom. *s* refers to a variable wrapped in a smooth function. Values in bold are statistically significant (*p* <0.05). | | | | |
| --- | --- | --- | --- | --- |
| Parametric coefficients | Estimate | Standard error | *t*-value | *p*-value |
| Intercept (*B. hortorum*: drone) | -3.31 | 0.0429 | -77.2 | **<0.001** |
| *B. lapidarius* | 0.0957 | 0.0484 | 1.98 | **0.0487** |
| *B. muscorum* | -0.0122 | 0.0633 | -0.193 | 0.847 |
| *B. pascuorum* | 0.111 | 0.0464 | 2.39 | **0.0173** |
| caste (queen) | 0.0569 | 0.0490 | 1.16 | 0.246 |
| caste (worker) | 0.112 | 0.0382 | 2.95 | **0.00334** |
|  |  |  |  |  |
| Smooth terms | EDF | DF | *F*-value | *p*-value |
| *s*(longitude, latitude) | 10.7 | 14.3 | 1.71 | **0.0495** |
| s(year, as a factor) | 29.8 | 87.0 | 0.603 | **<0.001** |

| Supplementary Table 7: ***F*-test comparing generalised additive mixed models with and without species-level intercepts, to** **estimate whether species differ in their baseline levels of fluctuating asymmetry*.*** DF = degrees of freedom; *N* = 587. | | | | | | | |
| --- | --- | --- | --- | --- | --- | --- | --- |
|  | Model DF | Residual  deviance | |  | Deviance | *F*-value | *p*-value |
| Model with species intercepts | 46.5 | 85.0 |  | |  |  |  |
| Model without species intercepts | 44.2 | 86.0 |  | | -0.962 | 2.58 | 0.067 |

| Supplementary Table 8: **Generalised additive model estimates to predict whether any of the species were disproportionately affected over the 20^th^ century.** The response variable was (logged) wing shape fluctuating asymmetry. R^2^ = 0.087, *N* = 587. EDF = effective degrees of freedom. *s* refers to a variable wrapped in a smooth function. Values in bold are statistically significant (*p* <0.05). | | | | |
| --- | --- | --- | --- | --- |
| Parametric coefficients | Estimate | Standard error | *t*-value | *p*-value |
| Intercept (*B. hortorum*: 1900-1949: drone) | -3.36 | 0.0533 | -63.0 | **<0.001** |
| *B. lapidarius* | 0.0776 | 0.0655 | 1.18 | 0.237 |
| *B. muscorum* | 0.0363 | 0.0851 | 0.427 | 0.669 |
| *B. pascuorum* | 0.123 | 0.0636 | 1.93 | **0.0542** |
| 1950-2000 | 0.0890 | 0.0742 | 1.20 | 0.238 |
| Caste (queen) | 0.0464 | 0.0498 | 0.932 | 0.352 |
| Caste (worker) | 0.103 | 0.0387 | 2.66 | **0.00809** |
| *B. lapidarius*: 1950-2000 | 0.0289 | 0.0964 | 0.300 | 0.764 |
| *B. muscorum*: 1950-2000 | -0.100 | 0.124 | -0.807 | 0.420 |
| *B. pascuorum*: 1950-2000 | -0.0336 | 0.0938 | -0.358 | 0.720 |
|  |  |  |  |  |
| Smooth terms | EDF | DF | *F*-value | *p*-value |
| *s*(longitude, latitude) | 12.3 | 16.5 | 2.32 | **0.00222** |

| Supplementary Table 9: ***F*-test comparing generalised additive models with and without an interaction between species-level intercepts and century half, to** **whether any species was disproportionately affected by environmental change over the two halves of the 20^th^ century** (1900-1949 vs. 1950-2000), as measured by (logged) wing shape fluctuating asymmetry. DF = degrees of freedom; *N* = 587. Values in bold are statistically significant (*p* <0.05). | | | | | | | |
| --- | --- | --- | --- | --- | --- | --- | --- |
|  | Model DF | Residual  deviance | |  | Deviance | *F*-value | *p*-value |
| Model with species × century half interaction | 22.3 | 96.1 |  | |  |  |  |
| Model without species × century half interaction | 19.2 | 96.4 |  | | -0.282 | 0.541 | 0.658 |

| Supplementary Table 10: **Generalised additive model estimates to assess difference in fluctuating asymmetry between the first and second halves of the 20^th^ century.** The response variable was (logged) wing shape fluctuating asymmetry. R^2^ = 0.090, *N* = 587. EDF = effective degrees of freedom. *s* refers to a variable wrapped in a smooth function. Values in bold are statistically significant (*p* <0.05). | | | | |
| --- | --- | --- | --- | --- |
| Parametric coefficients | Estimate | Standard error | *t*-value | *p*-value |
| Intercept (*B. hortorum,* 1900-1949) | -3.35 | 0.0438 | -76.5 | **<0.001** |
| *B. lapidarius* | 0.0904 | 0.0489 | 1.85 | 0.0650 |
| *B. muscorum* | -0.00820 | 0.0646 | -0.127 | 0.899 |
| *B. pascuorum* | 0.107 | 0.0470 | 2.27 | **0.0237** |
| 1950-2000 | 0.0752 | 0.0375 | 2.01 | **0.0452** |
| Caste (queen) | 0.0419 | 0.0496 | 0.846 | **0.398** |
| Caste (worker) | 0.103 | 0.0386 | 2.66 | **0.00805** |
|  |  |  |  |  |
| Smooth terms | EDF | DF | *F*-value | *p*-value |
| *s*(longitude, latitude) | 12.2 | 16.4 | 2.30 | **0.00255** |

| Supplementary Table 11: ***F*-test comparing generalised additive models with and without an intercept for each “century half” of the 20^th^ century, to estimate whether the two century halves (1900-1949 vs. 1950-2000) are statistically different in (logged) wing shape fluctuating asymmetry.** DF = degrees of freedom; *N* = 587. Values in bold are statistically significant (*p* <0.05). | | | | | | | |
| --- | --- | --- | --- | --- | --- | --- | --- |
|  | Model DF | Residual  deviance | |  | Deviance | *F*-value | *p*-value |
| Model with “century half” | 16.9 | 97.2 |  | |  |  |  |
| Model without “century half” | 16.6 | 97.2 |  | | -0.340 | 7.40 | **0.0338** |

| Supplementary Table 12: **Generalised additive model estimates for the change in (logged) wing shape fluctuating asymmetry over the 20^th^ century, when species are given their own intercepts and smooths over time.** *N* = 590; R^2^ = 0.111; AIC = 640.5 EDF = effective degrees of freedom. *s* refers to a variable wrapped in a smooth function. Values in bold are statistically significant (*p* <0.05). | | | | |
| --- | --- | --- | --- | --- |
| Parametric coefficients | Estimate | Standard error | *t*-value | *p*-value |
| Intercept (*B. hortorum*: drone) | -3.31 | 0.0401 | -82.4 | **<0.001** |
| *B. lapidarius* | 0.0688 | 0.0490 | 1.41 | 0.160 |
| *B. muscorum* | -0.000861 | 0.0642 | -0.134 | 0.893 |
| *B. pascuorum* | 0.103 | 0.0466 | 2.22 | **0.0272** |
| Caste (queen) | 0.0370 | 0.0492 | 0.751 | 0.453 |
| Caste (worker) | 0.0919 | 0.0383 | 2.40 | **0.0169** |
|  |  |  |  |  |
| Smooth terms | EDF | DF | *F*-value | *p*-value |
| *s*(year (scaled)): *B. hortorum* | 1.00 | 1.00 | 2.22 | 0.137 |
| *s*(year (scaled)): *B. lapidarius* | 2.86 | 3.56 | 3.96 | **0.00503** |
| *s*(year (scaled)): *B. muscorum* | 1.00 | 1.00 | 0.020 | 0.815 |
| *s*(year (scaled)): *B. pascuorum* | 1.00 | 1.00 | 2.46 | 0.163 |
| *s*(longitude, latitude) | 12.8 | 17.1 | 2.29 | **0.00221** |

| Supplementary Table 13: **Generalised additive model estimates for the change in (logged) wing shape fluctuating asymmetry over the 20^th^ century, when species are given their intercepts but a common smooth.** *N* = 590; R^2^ = 0.111; AIC = 639.6 EDF = effective degrees of freedom. *s* refers to a variable wrapped in a smooth function. Values in bold are statistically significant (*p* <0.05). | | | | |
| --- | --- | --- | --- | --- |
| Parametric coefficients | Estimate | Standard error | *t*-value | *p*-value |
| Intercept (*B. hortorum*: drone) | -3.31 | 0.0402 | -82.2 | **<0.001** |
| *B. lapidarius* | 0.0711 | 0.0488 | 1.46 | 0.145 |
| *B. muscorum* | -0.00302 | 0.0639 | -0.047 | 0.962 |
| *B. pascuorum* | 0.100 | 0.0466 | 2.15 | **0.0317** |
| Caste (queen) | 0.0337 | 0.0491 | 0.686 | 0.493 |
| Caste (worker) | 0.102 | 0.0381 | 2.67 | **0.00779** |
|  |  |  |  |  |
| Smooth terms | EDF | DF | *F*-value | *p*-value |
| *s*(year (scaled)) | 3.36 | 4.19 | 3.81 | **0.00347** |
| *s*(longitude, latitude) | 12.7 | 17.0 | 2.33 | **0.00189** |

| Supplementary Table 14: **Generalised additive model estimates for the change in (logged) wing shape fluctuating asymmetry over the 20^th^ century.** *N* = 590; R^2^ = 0.11; AIC = 638.0 EDF = effective degrees of freedom. *s* refers to a variable wrapped in a smooth function. Values in bold are statistically significant (*p* <0.05). | | | | |
| --- | --- | --- | --- | --- |
| Parametric coefficients | Estimate | Standard error | *t*-value | *p*-value |
| Intercept (drone) | -3.25 | 0.0254 | -128 | **<0.001** |
| Caste (queen) | 0.0243 | 0.0490 | 0.496 | 0.620 |
| Caste (worker) | 0.102 | 0.0377 | 2.72 | **0.00678** |
|  |  |  |  |  |
| Smooth terms | EDF | DF | *F*-value | *p*-value |
| *s*(year (scaled)) | 3.65 | 4.52 | 4.03 | **0.00163** |
| *s*(longitude, latitude) | 13.3 | 17.6 | 2.56 | **0.000468** |

| Supplementary Table 15: **F tests to compare generalised additive models with species-specific smooths over the 20^th^ century (model in Table S12) and species-specific intercepts (models in Tables S12 & S13).** As the model without species-level smooths and intercepts (Table S14) was not significantly worse, this model was chosen. DF = degrees of freedom. | | | | | | |
| --- | --- | --- | --- | --- | --- | --- |
|  | Model DF | Residual  deviance |  | Deviance | *F*-value | *p*-value |
| Model in Table S12 | 24.7 | 93.3 |  |  |  |  |
| Model in Table S13 | 22.1 | 93.6 |  | -0.364 | 1.43 | 0.239 |
| Model in Table S14 | 19.9 | 94.1 |  | -0.490 | 1.37 | 0.255 |

| Supplementary Table 16: **Generalised additive mixed model estimates for the change in (logged) wing shape fluctuating asymmetry with mean annual temperature and precipitation, when species have their own intercepts and smooths for the interaction between temperature and precipitation.** R^2^ = 0.20, *N* = 548, AIC = 602.8. EDF = Effective degrees of freedom, MAT = mean annual temperature, MAP = mean annual precipitation. *s* refers to a variable wrapped in a smooth function; *te* refers to variables combined in a 2D tensor produce smooth. Values in bold are statistically significant (*p* <0.05). | | | | |
| --- | --- | --- | --- | --- |
| Parametric coefficients | Estimate | Standard error | *t*-value | *p*-value |
| Intercept (*B. hortorum*: drone) | -3.33 | 0.0446 | -74.6 | **<0.001** |
| *B. lapidarius* | 0.109 | 0.0530 | 2.06 | **0.0404** |
| *B. muscorum* | -0.0134 | 0.0668 | -0.201 | 0.841 |
| *B. pascuorum* | 0.128 | 0.0479 | 2.67 | **0.00786** |
| Caste (queen) | 0.0449 | 0.0505 | 0.890 | 0.374 |
| Caste (worker | 0.104 | 0.0396 | 2.64 | **0.00856** |
|  |  |  |  |  |
| Smooth terms | EDF | DF | *F*-value | *p*-value |
| *te*(MAT, MAP): *B. hortorum* | 5.73 | 6.63 | 2.48 | **0.0168** |
| *te*(MAT, MAP): *B. lapidarius* | 8.56 | 10.7 | 1.85 | **0.0377** |
| *te*(MAT, MAP): *B. muscorum* | 4.05 | 4.79 | 0.529 | 0.728 |
| *te*(MAT, MAP): *B. pascuorum* | 5.41 | 6.35 | 2.28 | **0.0359** |
| *s*(longitude, latitude) | 8.36 | 11.3 | 2.01 | **0.0237** |
| *s*(year, as factor) | 22.7 | 75.0 | 0.489 | **0.00213** |

| Supplementary Table 17: **Generalised additive mixed model estimates for the change in (logged) wing shape fluctuating asymmetry with mean annual temperature and precipitation, when species have their own intercepts.** R^2^ = 0.18, *N* = 548, AIC = 589.8. EDF = Effective degrees of freedom, MAT = mean annual temperature, MAP = mean annual precipitation. *s* refers to a variable wrapped in a smooth function; *te* refers to variables combined in a 2D tensor produce smooth. Values in bold are statistically significant (*p* <0.05). | | | | |
| --- | --- | --- | --- | --- |
| Parametric coefficients | Estimate | Standard error | *t*-value | *p*-value |
| Intercept (*B. hortorum*: drone) | -3.32 | 0.0434 | -76.5 | **<0.001** |
| *B. lapidarius* | 0.101 | 0.0499 | 2.03 | **0.0431** |
| *B. muscorum* | -0.0164 | 0.0646 | -0.253 | 0.800 |
| *B. pascuorum* | 0.113 | 0.0472 | 2.38 | **0.0175** |
| Caste (queen) | 0.0484 | 0.0505 | 0.960 | 0.338 |
| Caste (worker) | 0.110 | 0.0392 | 2.80 | **0.00528** |
|  |  |  |  |  |
| Smooth terms | EDF | DF | *F*-value | *p*-value |
| *te*(MAT, MAP) | 8.87 | 11.1 | 2.41 | **0.00614** |
| *s*(longitude, latitude) | 7.73 | 10.5 | 2.00 | **0.0279** |
| *s*(year, as factor) | 25.4 | 75.0 | 0.584 | **<0.001** |

| Supplementary Table 18: **Generalised additive mixed model estimates for the change in (logged) wing shape fluctuating asymmetry with mean annual temperature and precipitation.** R^2^ = 0.18, *N* = 548, AIC = 591.2. EDF = Effective degrees of freedom, MAT = mean annual temperature, MAP = mean annual precipitation. *s* refers to a variable wrapped in a smooth function; *te* refers to variables combined in a 2D tensor produce smooth. Values in bold are statistically significant (*p* <0.05). | | | | |
| --- | --- | --- | --- | --- |
| Parametric coefficients | Estimate | Standard error | *t*-value | *p*-value |
| Intercept (drone) | -3.25 | 0.0298 | -109 | **<0.001** |
| Caste (queen) | 0.0362 | 0.0505 | 0.718 | 0.473 |
| Caste (worker) | 0.111 | 0.0388 | 2.88 | **0.00418** |
|  |  |  |  |  |
| Smooth terms | EDF | DF | *F*-value | *p*-value |
| *te*(MAT, MAP) | 8.99 | 11.2 | 2.33 | **0.00806** |
| *s*(longitude, latitude) | 9.05 | 12.2 | 2.11 | **0.0134** |
| *s*(year, as factor) | 24.5 | 75.0 | 0.546 | **0.00105** |

| Supplementary Table 19: **Analysis of variances with *F-*tests to compare generalised additive mixed models with species-specific smooths over mean annual temperature & precipitation (model in Table S16) and species-specific intercepts (models in Tables S16 & S17).** As the model without species-level smooths and intercepts (Table S18) was not significantly worse, this model was chosen. DF = degrees of freedom. | | | | | | |
| --- | --- | --- | --- | --- | --- | --- |
|  | Model DF | Residual  deviance | DF | Deviance | *F*-value | *p*-value |
| Model in Table S16 | 60.8 | 73.7 |  |  |  |  |
| Model in Table S17 | 48.0 | 77.1 | -20.9 | -3.45 | 1.09 | 0.354 |
| Model in Table S18 | 45.5 | 78.1 | -2.63 | -0.997 | 2.51 | 0.0664 |

| Supplementary Table 20: **Generalised additive mixed model estimates for the change in (logged) wing shape fluctuating asymmetry with maximum annual temperature and precipitation.** R^2^ = 0.18, *N* = 548, AIC = 594.2. Maximum annual temperature (MaxAT) refers to the annual mean of daily maximum air temperature for each UK Met Office climate region. Annual precipitation (AP) refers to the annual total precipitation amount for each climate region. EDF = Effective degrees of freedom; *s* refers to a variable wrapped in a smooth function; *te* refers to variables combined in a 2D tensor produce smooth. Bolded values are statistically significant (*p* <0.05). This model is visualised in Fig. S7. | | | | |
| --- | --- | --- | --- | --- |
| Parametric coefficients | Estimate | Standard error | *t*-value | *p*-value |
| Intercept (drone) | -3.25 | 0.0300 | -108 | **<0.001** |
| Caste (queen) | 0.0350 | 0.0505 | 0.693 | 0.489 |
| Caste (worker) | 0.112 | 0.0389 | 2.89 | **0.00406** |
|  |  |  |  |  |
| Smooth terms | EDF | DF | *F*-value | *p*-value |
| *te*(MaxAT, AP) | 8.35 | 10.4 | 2.26 | **0.0124** |
| *s*(longitude, latitude) | 8.75 | 11.8 | 2.04 | **0.0189** |
| *s*(year, as factor) | 25.3 | 75.0 | 0.580 | **<0.001** |

| Supplementary Table 21: **Generalised additive mixed model estimates for the change in (logged) wing shape fluctuating asymmetry with minimum annual temperature and precipitation.** R^2^ = 0.18, *N* = 548, AIC = 591.3. Minimum annual temperature (MinAT) refers to the annual mean of daily minimum air temperature for each UK Met Office climate region. Annual precipitation (AP) refers to the annual total precipitation amount for each climate region. EDF = Effective degrees of freedom; *s* refers to a variable wrapped in a smooth function; *te* refers to variables combined in a 2D tensor produce smooth. Bolded values are statistically significant (*p* <0.05). This model is visualised in Fig. S8. | | | | |
| --- | --- | --- | --- | --- |
| Parametric coefficients | Estimate | Standard error | *t*-value | *p*-value |
| Intercept (drone) | -3.25 | 0.0296 | -110 | **<0.001** |
| Caste (queen) | 0.0395 | 0.0505 | 0.781 | 0.435 |
| Caste (worker) | 0.113 | 0.0388 | 2.91 | **0.00380** |
|  |  |  |  |  |
| Smooth terms | EDF | DF | *F*-value | *p*-value |
| *te*(MinAT, AP) | 9.20 | 11.3 | 2.39 | **0.00626** |
| *s*(longitude, latitude) | 8.92 | 12.0 | 2.08 | **0.0173** |
| *s*(year, as factor) | 23.7 | 75.0 | 0.517 | **0.00163** |

| Supplementary Table 22: **Generalised additive mixed model estimates for the change in (logged) wing shape fluctuating asymmetry with maximum spring temperature.** R^2^ = 0.16, *N* = 548, AIC = 597.4. Maximum spring temperature (MaxSprT) refers to the mean of daily maximum air temperature for spring months (March, April & May) for each UK Met Office climate region. EDF = Effective degrees of freedom; *s* refers to a variable wrapped in a smooth function. Values in bold are statistically significant (*p* <0.05). This model is visualised in Fig. S9B. | | | | |
| --- | --- | --- | --- | --- |
| Parametric coefficients | Estimate | Standard error | *t*-value | *p*-value |
| Intercept (drone) | -3.25 | 0.0309 | -105 | **<0.001** |
| Caste (queen) | 0.0446 | 0.0510 | 0.874 | 0.382 |
| Caste (worker) | 0.121 | 0.0392 | 3.07 | **0.00223** |
|  |  |  |  |  |
| Smooth terms | EDF | DF | *F*-value | *p*-value |
| *s*(MaxSprT) | 1.00 | 1.00 | 1.53 | 0.217 |
| *s*(longitude, latitude) | 11.7 | 15.5 | 1.96 | **0.0156** |
| *s*(year, as factor) | 27.9 | 75.0 | 0.675 | **<0.001** |

| Supplementary Table 23: **Generalised additive mixed model estimates for the change in (logged) wing shape fluctuating asymmetry with minimum spring temperature.** R^2^ = 0.16, *N* = 548, AIC = 598.5. Minimum spring temperature (MinSprT) refers to the mean of daily minimum air temperature for spring months (March, April & May) for each UK Met Office climate region. EDF = Effective degrees of freedom; *s* refers to a variable wrapped in a smooth function. Values in bold are statistically significant (*p* <0.05). This model is visualised in Fig. S9A. | | | | |
| --- | --- | --- | --- | --- |
| Parametric coefficients | Estimate | Standard error | *t*-value | *p*-value |
| Intercept (drone) | -3.27 | 0.113 | -28.9 | **<0.001** |
| Caste (queen) | 0.0425 | 0.0510 | 0.832 | 0.406 |
| Caste (worker) | 0.120 | 0.0393 | 3.05 | **0.00238** |
| MinSprT | 0.00663 | 0.0306 | 0.216 | 0.829 |
|  |  |  |  |  |
| Smooth terms | EDF | DF | *F*-value | *p*-value |
| *s*(longitude, latitude) | 11.7 | 15.4 | 1.85 | **0.0253** |
| *s*(year, as factor) | 28.4 | 75.0 | 0.690 | **<0.001** |

| Supplementary Table 24: **Generalised additive mixed model estimates for the change in (logged) wing shape fluctuating asymmetry with spring precipitation.** R^2^ = 0.16, *N* = 548, AIC = 597.7. Spring precipitation (SprP) refers to the total precipitation amount for spring months (March, April & May) for each climate region. EDF = Effective degrees of freedom; *s* refers to a variable wrapped in a smooth function. Values in bold are statistically significant (*p* <0.05). This model is visualised in Fig. S9C. | | | | |
| --- | --- | --- | --- | --- |
| Parametric coefficients | Estimate | Standard error | *t*-value | *p*-value |
| Intercept (drone) | -3.18 | 0.0758 | -41.9 | **<0.001** |
| Caste (queen) | 0.0397 | 0.0510 | 0.780 | 0.436 |
| Caste (worker) | 0.120 | 0.0392 | 3.06 | **0.00232** |
| SprP | -0.000372 | 0.000339 | -1.10 | 0.273 |
|  |  |  |  |  |
| Smooth terms | EDF | DF | *F*-value | *p*-value |
| *s*(longitude, latitude) | 11.1 | 14.8 | 1.92 | **0.0194** |
| *s*(year, as factor) | 29.2 | 75.0 | 0.723 | **<0.001** |

| Supplementary Table 25: **Generalised additive mixed model estimates for the change in (logged) wing shape fluctuating asymmetry with maximum summer temperature.** R^2^ = 0.16, *N* = 548, AIC = 596.8. Maximum summer temperature (MaxSumT) refers to the mean of daily maximum air temperature for summer months (June, July & August) for each UK Met Office climate region. We note that 73% of specimens analysed here were collected during the summer months. EDF = Effective degrees of freedom; *s* refers to a variable wrapped in a smooth function. Values in bold are statistically significant (*p* <0.05). This model is visualised in Fig. S10B. | | | | |
| --- | --- | --- | --- | --- |
| Parametric coefficients | Estimate | Standard error | *t*-value | *p*-value |
| Intercept (drone) | -3.93 | 0.367 | -10.7 | **<0.001** |
| Caste (queen) | 0.0400 | 0.0509 | 0.787 | 0.432 |
| Caste (worker) | 0.115 | 0.0392 | 2.94 | **0.00348** |
| MaxSumT | 0.0364 | 0.0195 | 1.86 | 0.0632 |
|  |  |  |  |  |
| Smooth terms | EDF | DF | *F*-value | *p*-value |
| *s*(longitude, latitude) | 11.6 | 15.4 | 2.11 | **0.00845** |
| *s*(year, as factor) | 26.9 | 75.0 | 0.632 | **<0.001** |

| Supplementary Table 26: **Generalised additive mixed model estimates for the change in (logged) wing shape fluctuating asymmetry with minimum summer temperature.** R^2^ = 0.16, *N* = 548, AIC = 594.2. Minimum summer temperature (MaxSumT) refers to the mean of daily maximum air temperature for summer months (June, July & August) for each UK Met Office climate region. We note that 73% of specimens analysed here were collected during the summer months. EDF = Effective degrees of freedom; *s* refers to a variable wrapped in a smooth function. Values in bold are statistically significant (*p* <0.05). This model is visualised in Fig. S10A. | | | | |
| --- | --- | --- | --- | --- |
| Parametric coefficients | Estimate | Standard error | *t*-value | *p*-value |
| Intercept (drone) | -4.09 | 0.352 | -11.6 | **<0.001** |
| Caste (queen) | 0.0352 | 0.0508 | 0.693 | 0.489 |
| Caste (worker) | 0.111 | 0.0392 | 2.83 | **0.00486** |
| MinSumT | 0.0842 | 0.0354 | 2.38 | **0.0176** |
|  |  |  |  |  |
| Smooth terms | EDF | DF | *F*-value | *p*-value |
| *s*(longitude, latitude) | 13.1 | 17.1 | 2.26 | **0.00284** |
| *s*(year, as factor) | 24.9 | 75.0 | 0.553 | **0.00135** |

| Supplementary Table 27: **Generalised additive mixed model estimates for the change in (logged) wing shape fluctuating asymmetry with summer precipitation.** R^2^ = 0.16, *N* = 548, AIC = 596.0. Summer precipitation (SumP) refers to the total precipitation amount for summer months (June, July & August) for each climate region. We note that 73% of specimens analysed here were collected during the summer months. EDF = Effective degrees of freedom; *s* refers to a variable wrapped in a smooth function. Values in bold are statistically significant (*p* <0.05). This model is visualised in Fig. S10C. | | | | |
| --- | --- | --- | --- | --- |
| Parametric coefficients | Estimate | Standard error | *t*-value | *p*-value |
| Intercept (drone) | -3.08 | 0.0844 | -36.5 | **<0.001** |
| Caste (queen) | 0.0433 | 0.0508 | 0.852 | 0.395 |
| Caste (worker) | 0.120 | 0.0391 | 3.06 | **0.00232** |
| SumP | -0.000726 | 0.000341 | -2.13 | **0.0335** |
|  |  |  |  |  |
| Smooth terms | EDF | DF | *F*-value | *p*-value |
| *s*(longitude, latitude) | 10.8 | 14.4 | 2.17 | **0.00800** |
| *s*(year, as factor) | 28.2 | 75.0 | 0.689 | **<0.001** |

**SUPPLEMENTARY FIGURES**

**
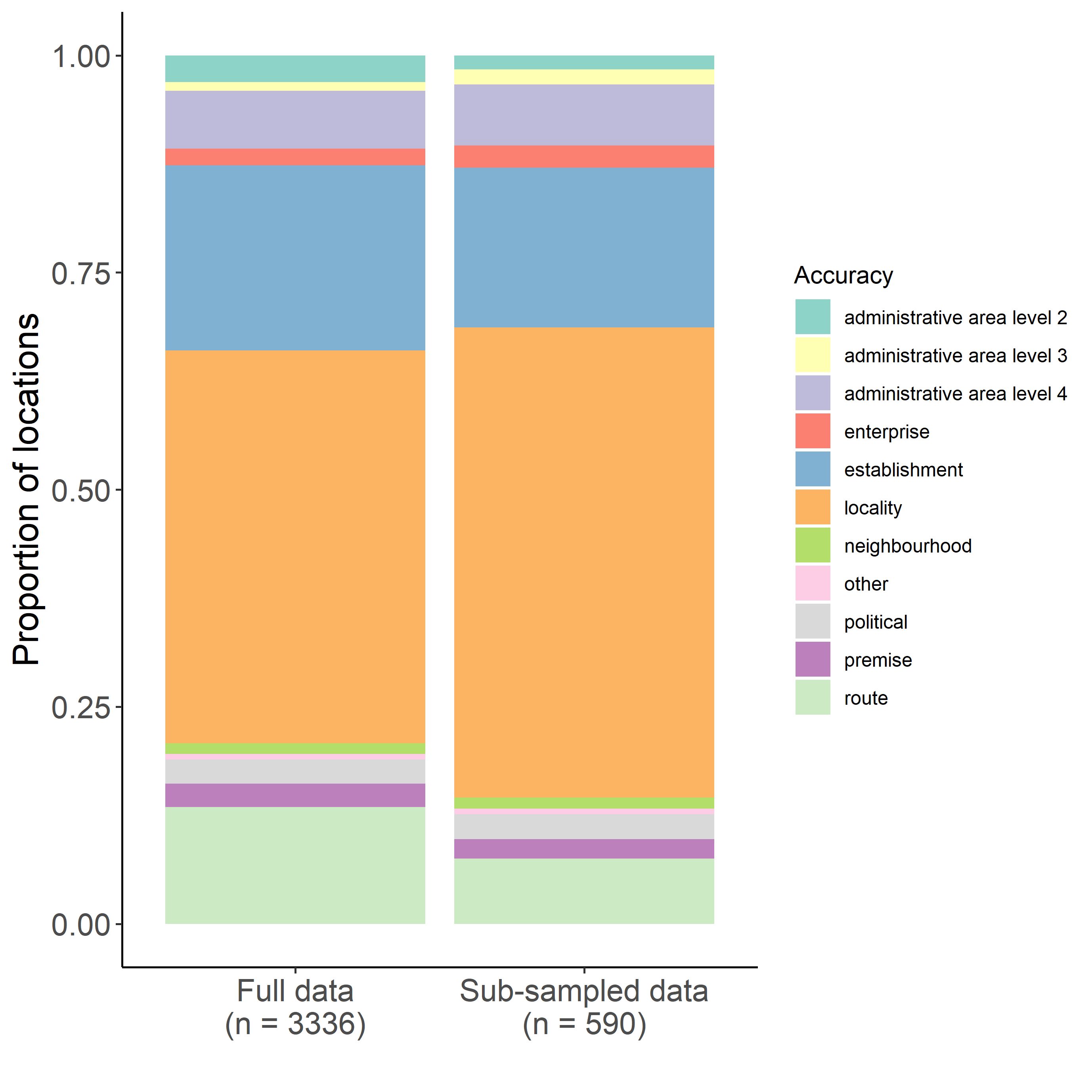
**

Supplementary Figure 1: **Types of location available on bumblebee specimen labels.** Accuracy refers to the type of location according to Google Maps’ Geocoding documentation, extracted using the “ggmap” package (Kahle & Wickham, 2013). “Administrative” areas refer to civil entities below the country level, with level four being the most resolved of the administrative areas used here. The category “enterprise” groups “airport”, “art gallery”, “bar”, “book store”, “bus station”, “café”, “electronics store” and “campground”. “Establishment” indicates a place that has not yet been categorised. “Locality” refers to a city or town. “Neighbourhood” indicates a named neighbourhood. “Political” refers to a political entity (usually a civil administration). “Premise” means a named location (usually a building). “Route” indicates a named route. “Other” contains the categories “archipelago”, “country”, “postal code”, “street address”, “postal town” and “administrative area level 1”, which were grouped due to their small representation among the bumblebee specimens. For more information please see: <https://developers.google.com/maps/documentation/geocoding/overview#Types>. Full data refers to the bumblebee specimens used before quality control and rarefaction (see main manuscript section 2.4). Subsampled data refers to the final dataset used in the main manuscript.

**
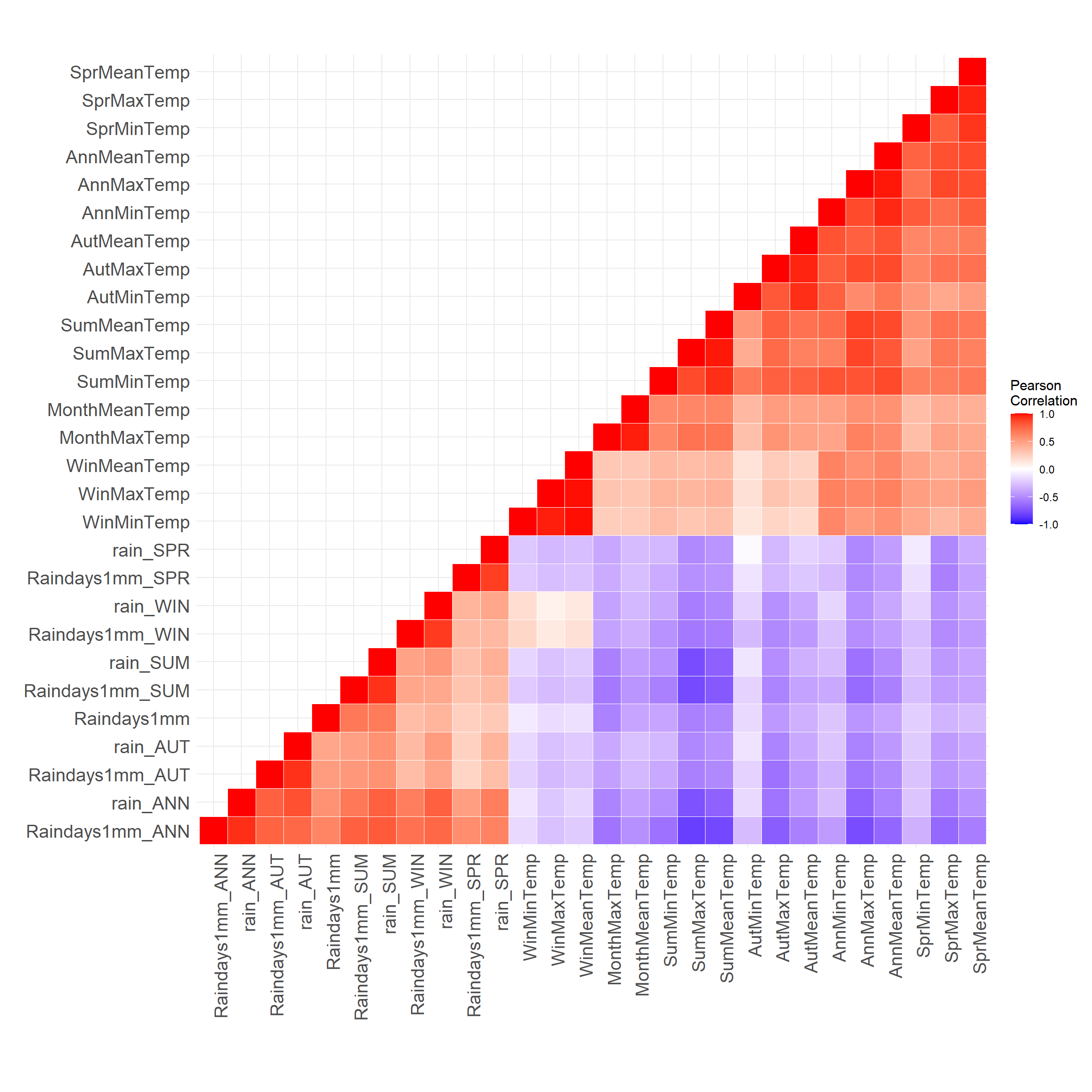
**

Supplementary figure 2: **Heatmap showing correlation among the climate variables accessed from UK Met Office.** Colour represents Pearson’s correlation, with red indicating a positive correlation, and blue, a negative. SUM = summer (June-August), AUT = autumn (September-November), WIN = winter (December-February), and SPR = spring (March-May).


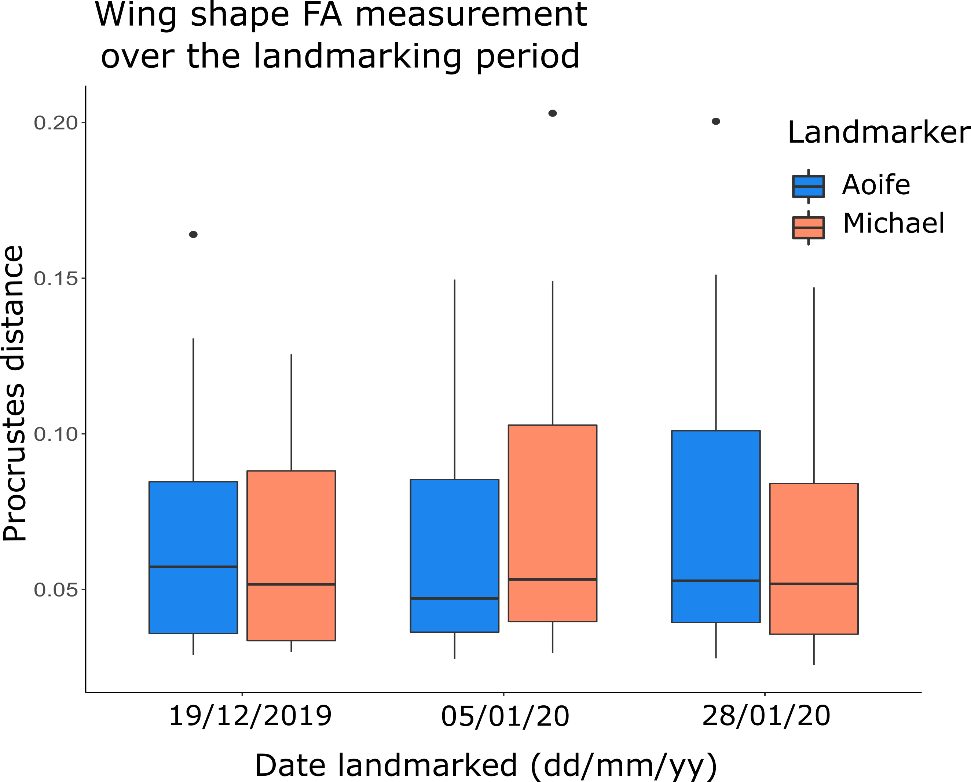


Supplementary figure 3: **Measurement of shape fluctuating asymmetry (FA) over the landmarking period.** To ensure consistent landmarking, 20 images (*B. hortorum* drones) were landmarked every three weeks over the digitisation period. Linear mixed-effects models were used to ensure no significant (*p*>0.05) differences in wing shape FA (the response variables) for each specimen (the random effect) occurred over the landmarking period (the fixed effect) or due to the person who performed the landmarking (fixed effect).

**
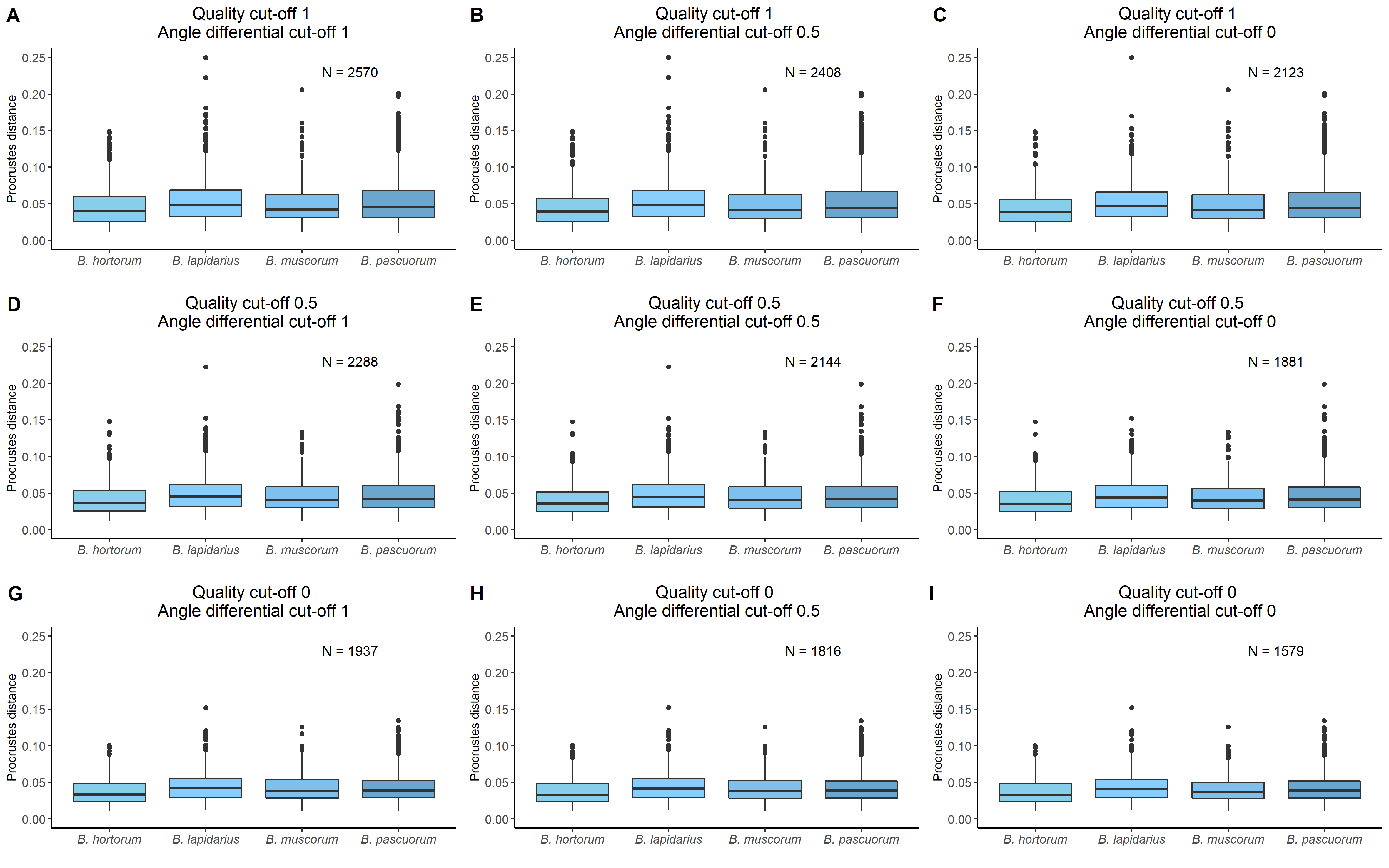
**

Supplementary Figure 4: **Changes in the distribution of wing-shape fluctuating asymmetry (measured using Procrustes distance) for each of the bumblebee species under different quality-control thresholds.** From top to bottom represents more stringent filtering of “wing quality”, i.e. Procrustes distance to the mean wing shape. From left to right represents more stringent filtering of individuals with forewings positioned at different angles. The quality-control thresholds used to generate plot G were selected to filter individuals in this study. “N” refers to the number of specimens remaining under those cut-offs.


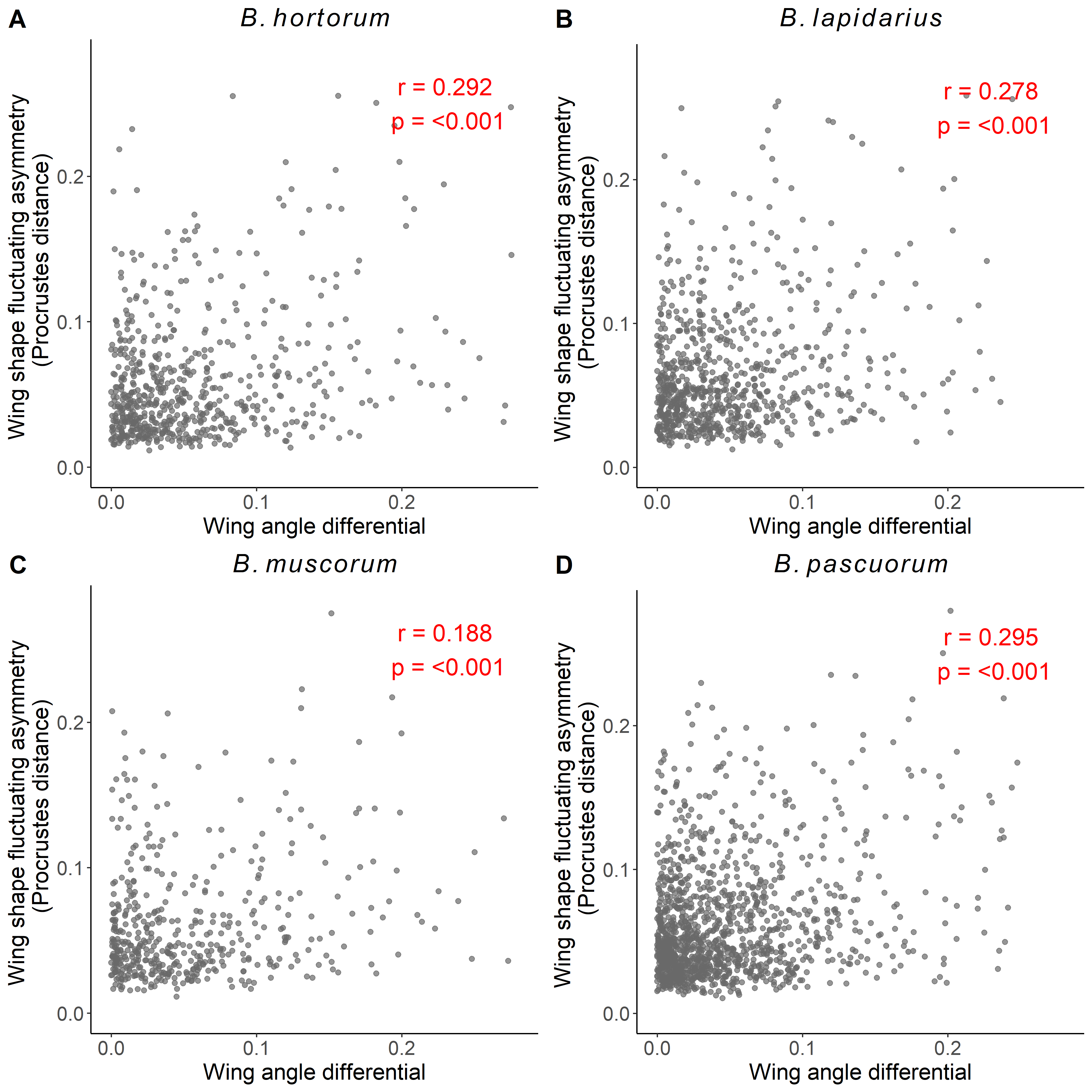


Supplementary Figure 5: **Correlation between wing angle differential and wing shape fluctuating asymmetry.** Wing angle differential refers to difference in the angle of the left and right forewings relative to the where each wing joins the specimen’s body. The correlations are based on data that was filtered to remove individuals with a wing shape or wing angle differential greater than three times the interquartile range to the mean wing shape or a differential of zero, respectively. A Pearson’s correlation test was used to assess correlation, with “r” referring to Pearson’s correlation coefficient and “p”, the *p*-value.

**
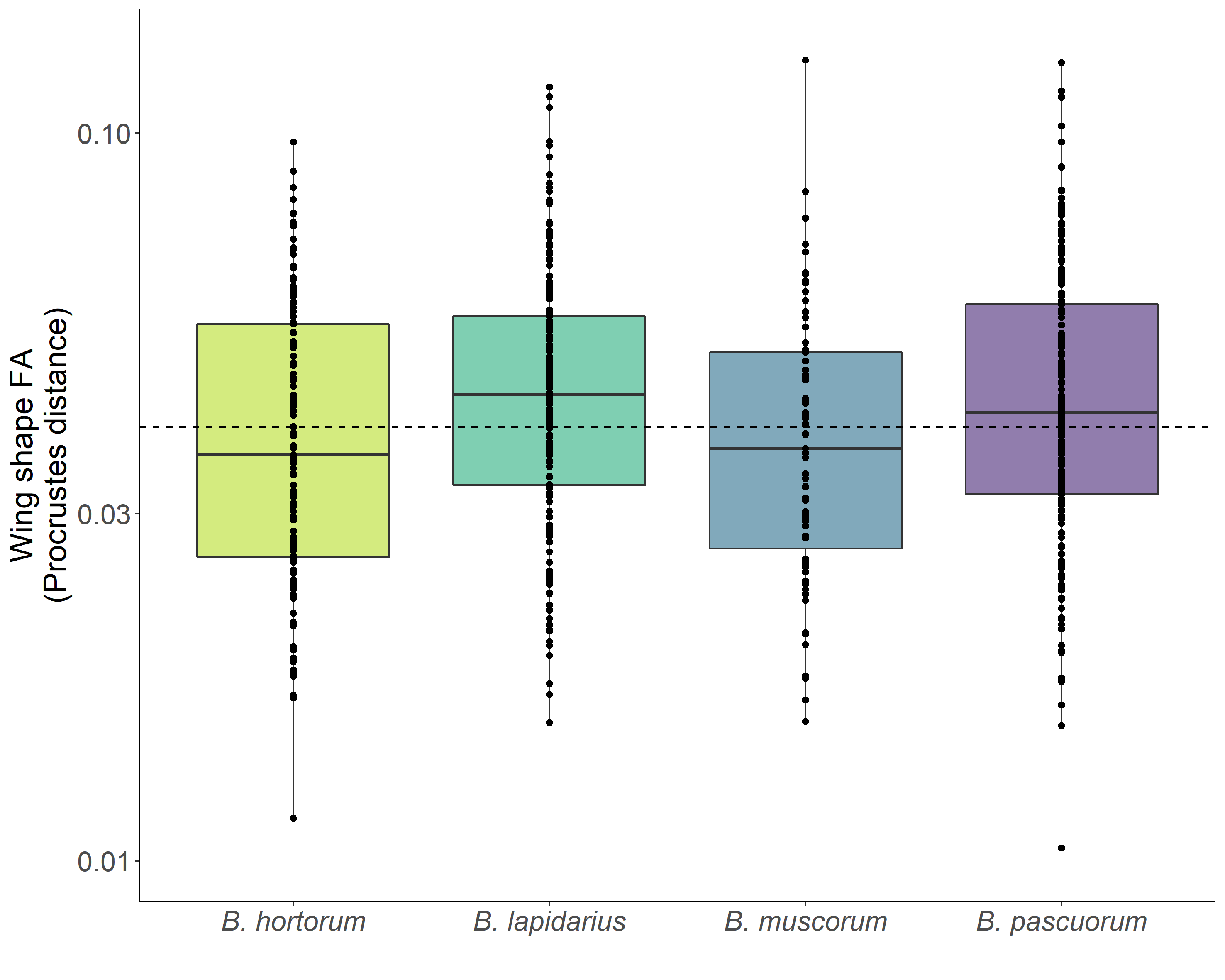
**

Supplementary Figure 6: **Bumblebee species differ in their baseline levels of wing shape fluctuating asymmetry (FA).** The dashed line represents the median value of the species-level medians. Individual points represent the raw FA values. The solid lines represent the median values for each species; the lower and upper bounds of each box, the 25^th^ and 75^th^ percentiles, respectively; and the lower and upper whiskers, the minimum and maximum values. FA values beyond the whiskers are deemed “outliers” (i.e. 1.5 × the interquartile range outside of the box). Note that the y-axis is on a log scale. Sample size of *B. hortorum* = 133, *B. lapidarius* = 179, *B. muscorum* = 75 and *B. pascuorum* = 203 specimens.


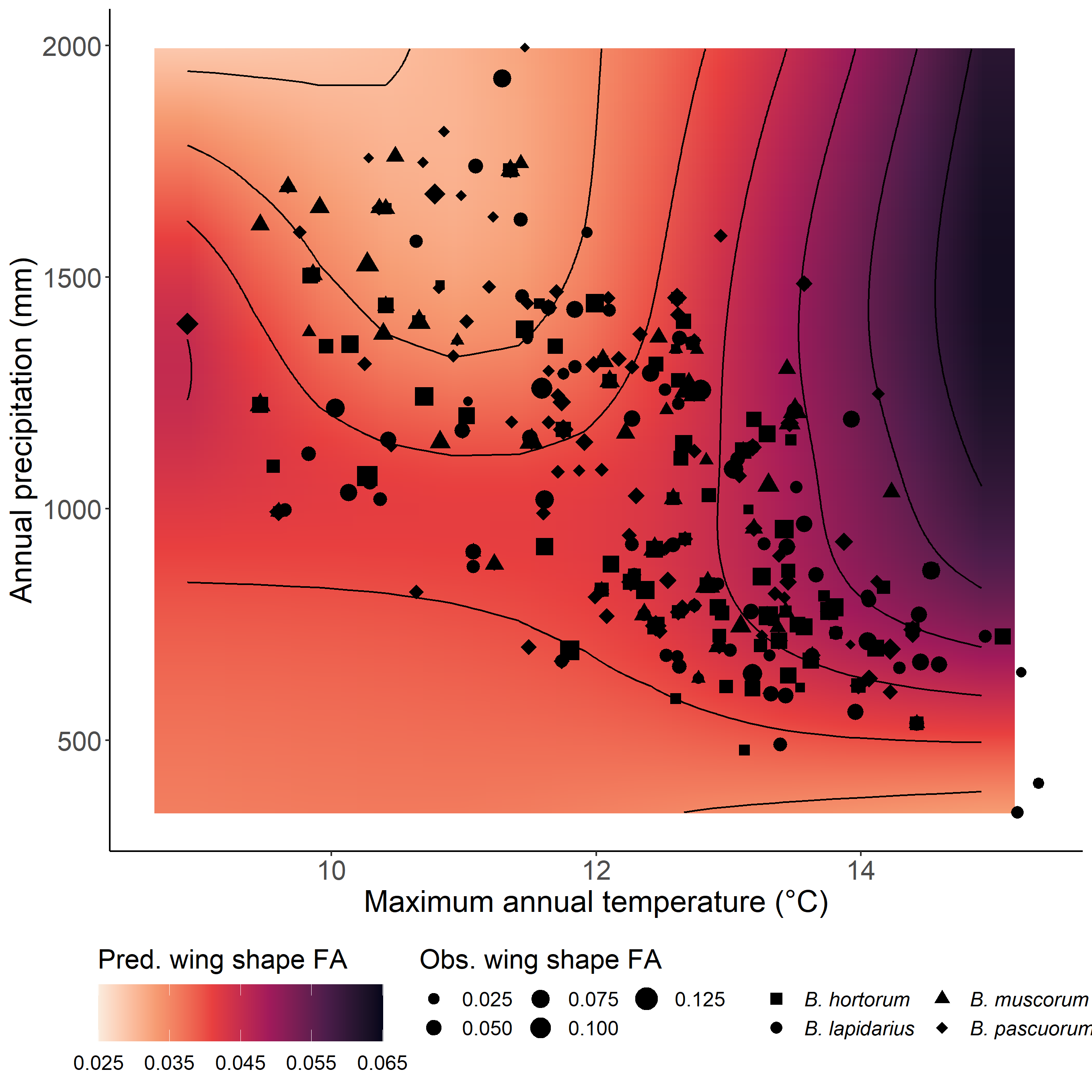


Supplementary Figure 7: **Bumblebee wing shape fluctuating asymmetry (FA) changes non-linearly with the combined effect of maximum annual temperature and precipitation.** Maximum annual temperature refers to the annual mean of daily maximum air temperatures for each UK Met Office climate region. Annual precipitation refers to the annual total precipitation amount for each climate region. Heatmap colours represent GAMM predictions of FA for workers sampled at mean latitude and longitude, as the GAMM used to predict FA accounted for variation across bumblebee castes and sample collection locations. Darker colours represent higher predicted (“Pred.”) FA*.* Points indicate the raw (“Obs.”) FA values, with the size indicating the magnitude and different shapes representing the four species.


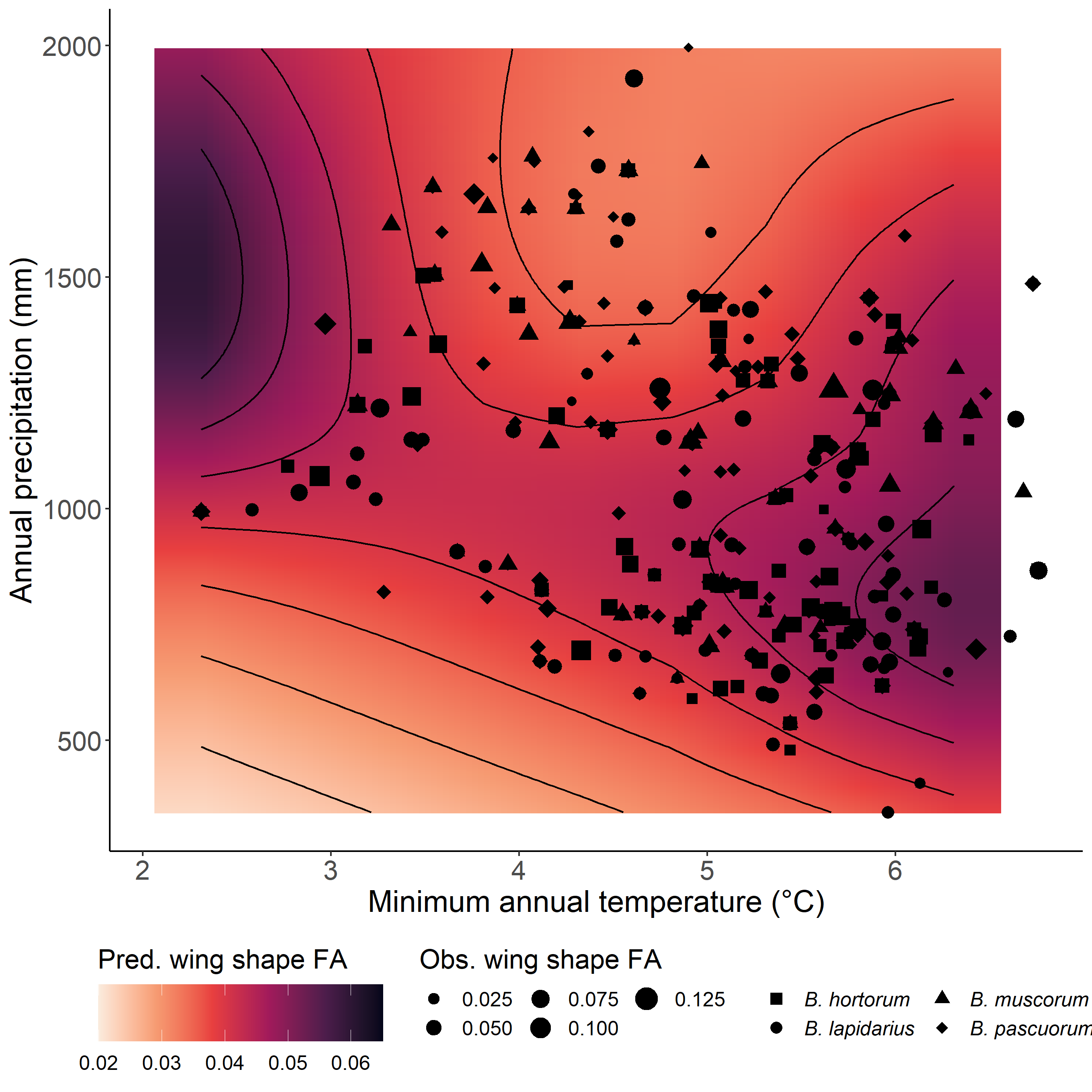


Supplementary Figure 8: **Bumblebee wing shape fluctuating asymmetry (FA) changes non-linearly with the combined effect of minimum annual temperature and precipitation.** Minimum annual temperature refers to the annual mean of daily minimum air temperatures for each UK Met Office climate region. Annual precipitation refers to the annual total precipitation amount for each climate region. Heatmap colours represent GAMM predictions of FA for workers sampled at mean latitude and longitude, as the GAMM used to predict FA accounted for variation across bumblebee castes and sample collection locations. Darker colours represent higher predicted (“Pred.”) FA*.* Points indicate the raw (“Obs.”) FA values, with the size indicating the magnitude and different shapes representing the four species.


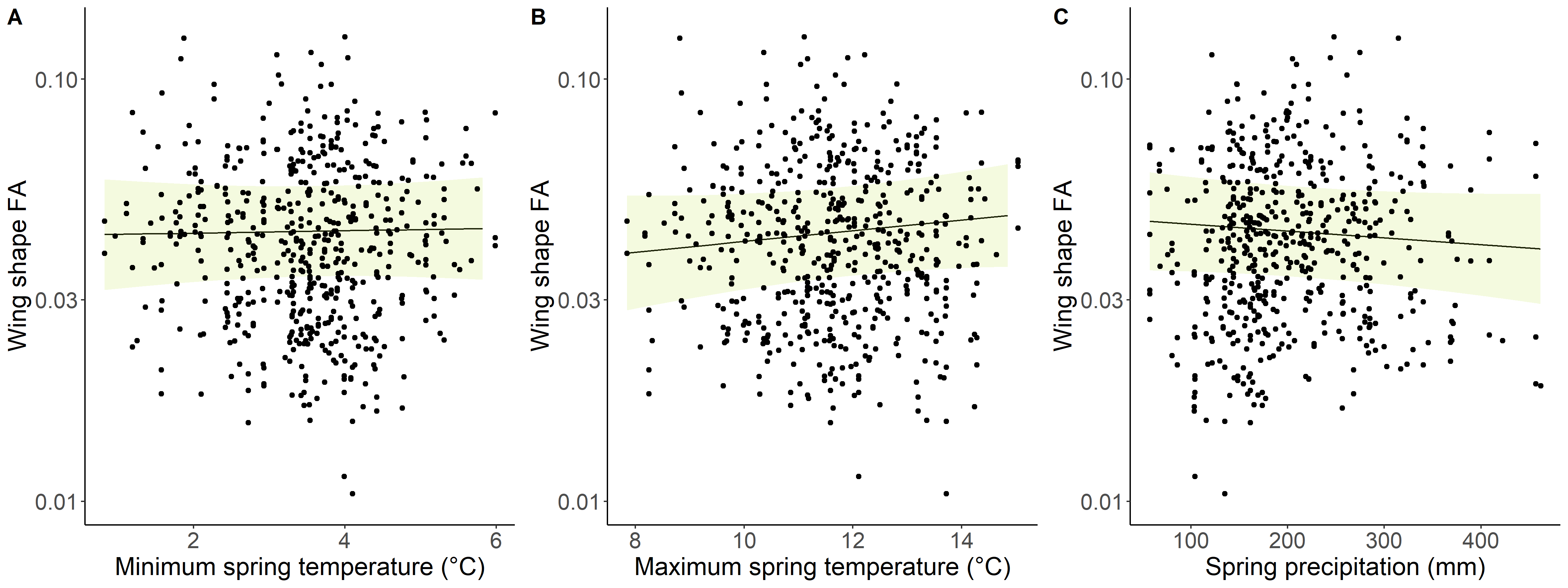


Supplementary Figure 9: **Relationship between bumblebee wing shape fluctuating asymmetry (FA) with spring A) minimum temperature, B) maximum temperature, and C) precipitation.** Spring encompasses March, April and May. Minimum and maximum temperatures refer to the mean of daily minimum and maximum air temperatures for each UK Met Office climate region during the spring period, respectively. Spring precipitation refers to the spring total precipitation amount for each climate region.


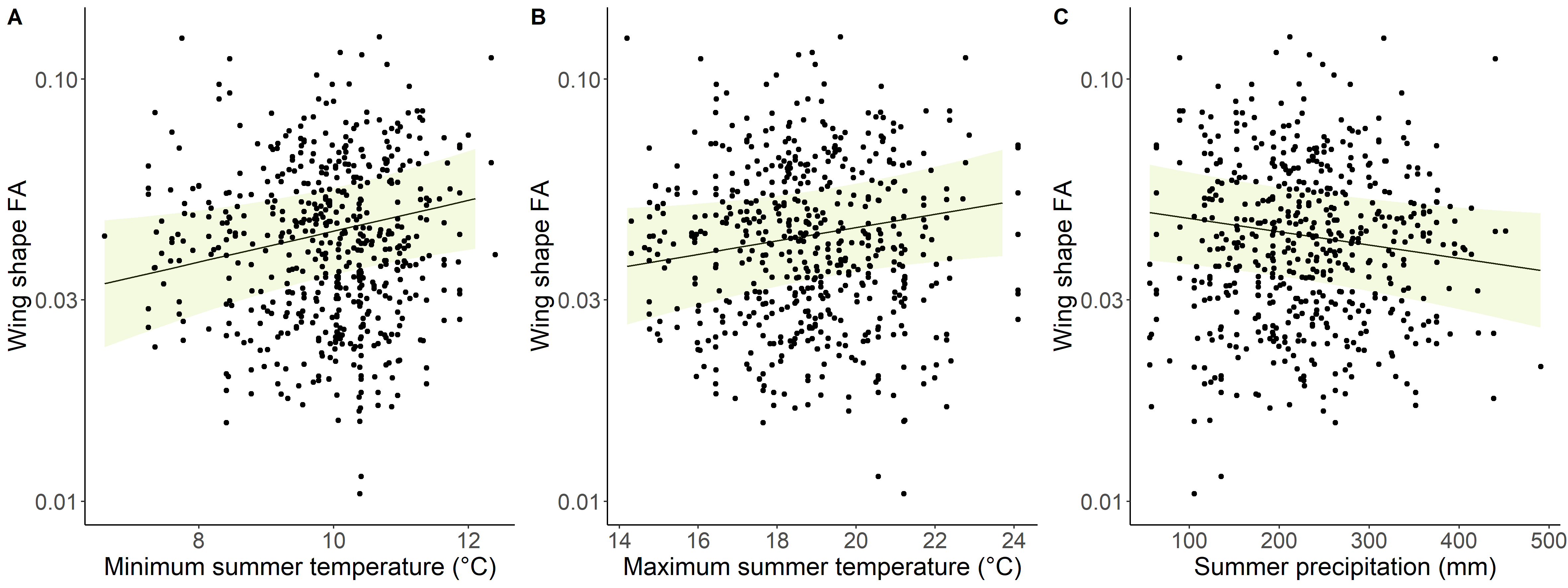


Supplementary Figure 10: **Relationship between bumblebee wing shape fluctuating asymmetry (FA) with summer A) minimum temperature, B) maximum temperature, and C) precipitation.** Summer encompasses June, July and August. We note that 73% of specimens analysed here were collected during these months. Minimum and maximum temperatures refer to the mean of daily minimum and maximum air temperatures for each UK Met Office climate region during the summer period, respectively. Summer precipitation refers to the summer total precipitation amount for each climate region.

**SUPPLEMENTARY REFERENCES**

Duchenne, F., Thébault, E., Michez, D., Elias, M., et al. (2020) Phenological shifts alter the seasonal structure of pollinator assemblages in Europe. *Nature Ecology and Evolution.* *4(1)*, 115-121.

Friedli, A., Williams, G.R., Bruckner, S., Neumann, P., et al. (2020) The weakest link: Haploid honey bees are more susceptible to neonicotinoid insecticides. *Chemosphere*. *242*, 125145.

Graham, J.H., Raz, S., Hel-Or, H. & Nevo, E. (2010) Fluctuating asymmetry: methods, theory, and applications. *Symmetry*. *2(2)*, 466-540.

Kahle, D.J. & Wickham, H. (2013) ggmap: spatial visualization with ggplot2. *The* *R Journal.* *5(1)*, 144.

Kark, S. (2001) Shifts in bilateral asymmetry within a distribution range: the case of the chukar partridge. *Evolution*. *55(10)*, 2088-2096.

Scrucca, L., Fop, M., Murphy, T.B. & Raftery, A.E. (2016) mclust 5: clustering, classification and density estimation using Gaussian finite mixture models. *The R journal*. *8(1)*, 289.
